# Caspase vinyl sulfone small molecule inhibitors prevent axonal degeneration in human neurons and reverse cognitive impairment in Caspase-6-overexpressing mice

**DOI:** 10.1186/s13024-017-0166-z

**Published:** 2017-02-28

**Authors:** Prateep Pakavathkumar, Anastasia Noël, Clotilde Lecrux, Agne Tubeleviciute-Aydin, Edith Hamel, Jan-Eric Ahlfors, Andrea C. LeBlanc

**Affiliations:** 1 Bloomfield Center for Research in Aging, Lady Davis Institute for Medical Research, Jewish General Hospital, 3999 Ch. Cote Ste-Catherine, Montreal, QC H3T 1E2 Canada; 2 Department of Neurology and Neurosurgery, McGill University, 845 Sherbrooke O, Montreal, QC H3A 0G4 Canada; 3 Laboratory of Cerebrovascular Research, Montreal Neurological Institute, 3801 University Street, Montreal, QC H3A 2B4 Canada; 4 New World Laboratories, 500 Boulevard Cartier Ouest, Laval, QC H7V 5B7 Canada; 5 Molecular and Regenerative Medicine Axis, Lady Davis Institute for Medical Research, Sir Mortimer B Davis Jewish General Hospital, 3755 ch. Côte Ste-Catherine, Montréal, QC H3T 1E2 Canada

**Keywords:** Alzheimer disease, Caspases, Caspase-6, Axonal degeneration, Peptide inhibitors, Primary human neurons, Caspase-6 transgenic mice, Vinyl sulfone inhibitors

## Abstract

**Background:**

The activation of the aspartate-specific cysteinyl protease, Caspase-6, is proposed as an early pathogenic event of Alzheimer disease (AD) and Huntington’s disease. Caspase-6 inhibitors could be useful against these neurodegenerative diseases but most Caspase-6 inhibitors have been exclusively studied in vitro or show acute liver toxicity in humans. Here, we assessed vinyl sulfone small molecule peptide caspase inhibitors for potential use in vivo.

**Methods:**

The IC_50_ of NWL vinyl sulfone small molecule caspase inhibitors were determined on Caspase-1 to 10, and Caspase-6-transfected human colon carcinoma HCT116 cells. Inhibition of Caspase-6-mediated axonal degeneration was assessed in serum-deprived or amyloid precursor protein-transfected primary human CNS neurons. Cellular toxicity was measured by phase contrast microscopy, mitochondrial and lactate dehydrogenase colorimetric activity assays, or flow cytometry. Caspase inhibition was measured by fluorogenic activity assays, fluorescence microscopy, and western blot analyses. The effect of inhibitors on age-dependent cognitive deficits in Caspase-6 transgenic mice was assessed by the novel object recognition task. Liquid chromatography coupled to tandem mass spectrometry assessed the blood-brain barrier permeability of inhibitors in Caspase-6 mice.

**Results:**

Vinyl sulfone NWL-117 caspase inhibitor has a higher selectivity against Caspase-6, −4, −8, −9, and −10 whereas NWL-154 has higher selectivity against Caspase-6, −8, and −10. The half-maximal inhibitory concentrations (IC_50_) of NWL-117 and NWL-154 is 192 nM and 100 nM against Caspase-6 in vitro, and 4.82 μM and 3.63 μM in Caspase-6-transfected HCT116 cells, respectively. NWL inhibitors are not toxic to HCT116 cells or to human primary neurons. NWL-117 and NWL-154 inhibit serum deprivation-induced Caspase-6 activity and prevent amyloid precursor protein-mediated neurite degeneration in human primary CNS neurons. NWL-117 crosses the blood brain barrier and reverses age-dependent episodic memory deficits in Caspase-6 mice.

**Conclusions:**

NWL peptidic vinyl methyl sulfone inhibitors are potent, non-toxic, blood-brain barrier permeable, and irreversible caspase inhibitors with neuroprotective effects in HCT116 cells, in primary human CNS neurons, and in Caspase-6 mice. These results highlight the therapeutic potential of vinyl sulfone inhibitors as caspase inhibitors against neurodegenerative diseases and sanction additional work to improve their selectivity against different caspases.

**Electronic supplementary material:**

The online version of this article (doi:10.1186/s13024-017-0166-z) contains supplementary material, which is available to authorized users.

## Background

Alzheimer disease (AD) is a neurodegenerative condition characterized by cognitive impairments leading to dementia with no disease-modifying treatments. Pathologically, AD is defined by an accumulation of extracellular plaques containing mostly amyloid-beta peptide (Aβ) and intracellular neurofibrillary tangles (NFT) composed of a hyper-phosphorylated form of the microtubule-associated protein Tau. Clinical trials targeting Aβ plaques have been unsuccessful in restoring cognitive function [1], while trials on disaggregating NFTs are currently ongoing [2]. The results from the current clinical trials suggest that therapeutic intervention against AD could be improved by targeting earlier pathogenic events.

One emerging potential disease-modifying therapeutic target is Caspase-6 (Casp6), a cysteinyl protease that cleaves protein substrates specifically after an aspartic acid residue [3]. Casp6, but not Casp3 or Casp7, is activated in neurites interspersing Aβ plaques, NFTs, and neuropil threads of familial and sporadic AD brains [4–6]. In brains from some aged non-cognitively impaired individuals, Casp6 activity levels correlates negatively with episodic and semantic memory performance [7], two types of memory first affected in AD. Tau-cleaved by Casp6 (TauΔCasp6) levels in post-mortem cerebrospinal fluid correlate inversely with episodic, semantic, and working memory performance [8]. Overexpression of human Casp6 in the CA1 region of mice hippocampi results in age-dependent episodic and spatial memory loss [9]. These findings suggest that early Casp6 activation in the hippocampus of aged pre-symptomatic individuals leads to cognitive impairment.

When activated, Casp6 can impair the microtubule network within neuronal axons and lead to degeneration. Casp6 cleaves the C-terminus of several neuronal cytoskeletal or associated proteins including Tau and α-tubulin [4, 10]. In human CNS neuron cultures, overexpression of wild type amyloid precursor protein (APP^WT^), a condition associated with familial AD [11], results in Casp6-dependent, but Aβ-independent, neuritic degeneration [12]. Therefore, inhibiting Casp6 activity could prevent axonal degeneration.

Caspases play an important role in other neurodegenerative conditions. Casp6 is associated with motor impairment in the Huntington mouse model [13–18]. Casp6-dependent tubulin fragmentation is associated with neuritic degeneration in mouse sympathetic, retinocollicular, dorsal root ganglion sensory, commissural, and motor neurons following nerve growth factor (NGF)-deprivation [19–21]. Casp6 participates in axonal degeneration of neurturin-deprived dorsal root ganglion cells [22], myelin-mediated sympathetic and septal cholinergic neurons [23], ischemic neurons [24, 25], and retinal ganglion cells in models of optic nerve injury in rodents [26]. In these conditions, Casp6 is activated in the presence of other caspases, including Casp9 and Casp3. Similarly, human neonatal, infant, and adult hypoxic-ischemic brain injury results in increased levels of active Casp6, active Casp3, and tubulin cleaved by Casp6/3 (TubΔCasp6/3) [4, 27].

Natural caspase inhibitors either do not inhibit Casp6 or are non-selective. Viral proteins p35 and CrmA inhibit several caspases [28, 29]. The mammalian inhibitors of apoptosis proteins (IAP) do not inhibit Casp6 [30, 31]. There are two natural Casp6 protein inhibitors: the alternatively spliced Casp6β isoform, which only prevents Casp6 activation and the caspase inhibitory factor (CIF), which is reactive against other caspases [32, 33]. Many competitive small molecule Casp6 inhibitors have been developed but most have not been tested for cellular toxicity, blood brain barrier permeability and in vivo inhibition. Aza-peptides specifically inhibit caspases and not other cysteine proteases [34]. Casp6 specificity is improved with sulfonamide isatin Michael acceptors [35]. Aldehyde or fluoromethyl ketones (fmk)-conjugated peptides obtained from positional scanning libraries or natural AP-2α and Lamin A Casp6 substrates have been used as Casp6 inhibitors [18, 36–41]. The commercially available Casp6 inhibitor benzyloxycarbonyl-Val-Glu-Ile-Asp-fmk (Z-VEID-fmk) is toxic to mammals because the fmk moiety can be metabolized into fluorocitrate, an inhibitor of aconitase that depletes tricarboxylic acid cycle intermediates [42]. Nevertheless, a Huntington-based peptide inhibitor conjugated to TAT to enhance membrane permeability and delivered to the brain with an osmotic pump protects against behavioral and motor deficits in a mutant Huntingtin mouse model [18].

New World Laboratories Inc. (NWL) has developed novel peptidomimetic irreversible small molecule inhibitors that retain Casp6’s Z-VEID preferred substrate, but have a methyl vinyl sulfone chemical warhead which 1) is selective for cysteinyl proteases, 2) is unreactive with circulating thiols or non-active site cysteines, 3) forms a hydrogen bond with the active site histidine [43], and (4) is safe in rats, dogs, and primates [44]. Here, we describe a non-toxic and blood-brain permeable NWL caspase inhibitor that prevents axonal degeneration of primary human neurons, and reverses Casp6-dependent episodic memory impairment in mice. These findings highlight vinyl sulfones as viable caspase inhibitors for pre-clinical studies.

## Methods

### DNA constructs

The mammalian constructs encoding human Casp6p20p10 in the pCep4β vector (Thermo Fisher Scientific, Waltham, MA, USA) [45], and enhanced green flurorescent protein (EGFP) or EGFP and amyloid precursor protein (APP^WT^) in the double promoter-containing pBudCE4.1 vector (Thermo Fisher Scientific, Waltham, MA, USA) [12] were previously cloned in our laboratory. A synthetic *Escherichia coli* codon-optimized gene (GenScript, Piscataway, NJ, USA) coding for human Casp6 large subunit (amino acids 24-179, flanked by start (ATG) and stop (TAA) codons) and small subunit (amino acids 194-293, preceded by a start codon), separated by GAATTCAATAATTTTGTTTAACTTTAAGAAGGAGATATACAT containing an internal ribosome binding site (underlined), was ligated into the XbaI/XhoI sites of the pET23b(+)-Casp6-His plasmid (a kind gift from Dr. Guy Salvesen, Sanford Burnham Prebys Medical Discovery Institute, CA, USA), under the control of a single T7 promoter. All plasmids were sequenced by the Sanger method (McGill University and Genome Quebec Innovation Center, Montreal, Quebec, CA).

*Recombinant Casp6 Expression and purification*: Casp6 was expressed from the pET23b(+)-Casp6-His plasmid in *E. coli* BL21(DE3)pLysS strain (Promega, Fitchburg, WI, USA) at 37 °C in 2xYT medium (16 g/l tryptone, 10 g/l yeast extract, 5 g/l NaCl) supplemented with 0.1 mg/ml ampicillin and 0.034 mg/ml chloramphenicol under vigorous shaking according to [46]. Casp6 expression was induced with 50 μM isopropyl β-D-1-thiogalactopyranoside (IPTG) when cell cultures reached OD_595 nm_ of 0.6 and cells cultured at 22 °C for 16 h under vigorous shaking. Cells were harvested by centrifugation, resuspended in buffer A (50 mM Tris pH 8.5, 300 mM NaCl, 5% glycerol, 2 mM imidazole), and lysed by sonicating on ice with a Vibra-Cell ultrasonic processor (Sonics and Materials, Newtown, CT, USA) for 2 min at 50% duty with output control set to four. The lysate was clarified by centrifugation (30,000 x *g* for 30 min at 4 °C) and loaded on Ni Sepharose Fast Flow 6 medium (GE Healthcare Life Sciences, Baie D’Urfe, QC, CA) pre-equilibrated with buffer A, washed with buffer B (50 mM Tris pH 8.5, 500 mM NaCl, 5% glycerol, 20 mM imidazole), and bound proteins eluted with a 50-300 mM linear imidazole gradient in buffer A. Fractions were assessed for recombinant Casp6 purity by SDS-PAGE and Coomassie blue staining. Fractions containing pure Casp6 were pooled together, dialyzed against storage buffer (20 mM Tris pH 8.5, 200 mM NaCl, 10 mM DTT, 5% glycerol), concentrated by dialysis against polyethylene glycol (PEG) 20,000 (Sigma-Aldrich, Oakville, ON, CA), and stored at -80 °C in small aliquots. Protein concentration was measured using Quick Start Bradford 1x Dye Reagent (Bio-Rad Laboratories, Hercules, CA, USA). *Active site titration assay*: The concentration of Casp6 active sites was determined by active site titration assay using Z-VAD-fmk (N-benzyloxycarbonyl-Val-Ala-Asp-(O-methyl)-fluoromethylketone, MP Biomedicals, Santa Ana, CA, USA) inhibitor [46]. Casp6 (398 nM) was incubated in SB with 0 to 1.25 μM Z-VAD-fmk for 2 h at room temperature in a final volume of 10 μl, diluted 20-fold with SB, and 25 μl of aliquots transferred to a black clear bottom 96-well microplate (Costar, Corning, NY, USA). Casp6 VEIDase activity (see below) was plotted as a function of Z-VAD-fmk concentration; the intersection at the X-axis in the linear region of the curve indicates the concentration of active sites of Casp6.

### Cell cultures and NWL inhibitor treatments

HCT116: Human colon carcinoma (HCT116) cells (ATCC, Manassas, VA, USA) were cultured in McCoy’s 5A modified media (Thermo Fisher Scientific, Waltham, MA, USA) supplemented with 10% fetal bovine serum (Thermo Fisher Scientific, Waltham, MA, USA) and transfected with 1 μg of pCep4βCasp6p20p10 mixed with 8 μg of polyethyleneimine (Polysciences Inc., Warrington, PA, USA) [47]. Protein expression was allowed for 24 h before treating with vehicle (PBS), 100 μM NWL-117, or 100 μM NWL-154 for 2 h. For NWL inhibitor kinetic experiments, 100 μM of NWL-117 or NWL-154 was added at 120, 90, 60, 30, 15, or 0 min before harvest. For the extended time course from 2 to 48 h, NWL inhibitors were dissolved in PEG400 (Sigma-Aldrich, Oakville, ON, CA) (50% v/v), anhydrous ethanol (20% v/v), and 154 mM NaCl (BioShop Canada Inc, Burlington, Ontario, CA) (30% v/v)). For recovery time course experiments, after the initial treatment with 100 μM of NWL-117 or −154 for 2 h, the media was replaced with fresh media for 120, 90, 60, 30, 15, or 0 min before harvest. Primary Human CNS neurons: Cortical tissues were obtained from the Birth Defects Research Laboratory (BDRL, University of Washington, Seattle, USA) in accordance with NIH ethical guidelines approved by McGill University’s institutional review board and primary neurons cultured as previously described [48]. Primary human neurons were seeded on poly-L-lysine-coated (5 μg/mL) 6-well plates or glass coverslips coated with poly-L-lysine and laminin (5 μg/mL) (Sigma-Aldrich, Oakville, ON, CA) at a density of 3x10^6^ cells/mL. Primary human neurons were pre-treated with 0.1 μM epoxomicin (Enzo LifeSciences, Farmingdale, NY, USA) and 100 μM NWL-117, 100 μM NWL-154, or vehicle (PBS) for 2 h, and serum-deprived for 2 h in the presence of epoxomicin and vehicle (PBS), 100 μM NWL-117, or 100 μM NWL-154 before harvesting or measuring caspase activity with FLICA, as described below.

### Casp6 activity assays

*Recombinant or extracted cellular Casp6 activity*: Casp6 activity was assessed by in vitro fluorogenic assays using Ac-Val-Glu-Ile-Asp-(7-Amino-4-trifluoromethylcouramin) (Ac-VEID-AFC: Enzo LifeSciences, NY, USA) as the Casp6 substrate. The activity was measured in Stennicke’s buffer (SB) (20 mM piperazine-N, N-bis (2-ethanesulfonic acid) (PIPES: BioShop Canada Inc, Burlington, Ontario, CA) pH 7.2, 30 mM NaCl, 1 mM ethylenediaminetetraacetic acid (EDTA), 0.1% 3-[(3-cholamidopropyl)-dimethylammonio]-2-hydroxy-1-propanesulfonic acid (CHAPS), 10% sucrose) [49]. The reaction mix consisted of either 20 nM RCasp6 or 20–30 μg cellular protein extracts, SB, 10 mM DTT, 10 μM VEID-AFC substrate and deionized water. The activity was measured in a black clear bottom 96-well plate (Costar, Corning, NY, USA) at 50 μL/well in triplicate at 37 °C in the Synergy H4 plate reader (BioTek) at excitation 380 nm and emission 505 nm every two minutes for 100 min. Fluorescence units were converted to the moles of AFC released based on a standard curve of 0–625 picomoles of free AFC. Cleavage rates were calculated from the linear phase of the assay. The activity is considered on a percentage scale where no inhibitor present is equated to 100% activity of the enzyme. *Cellular Casp6 activity assay by FLICA*: Active Casp6 was labeled within primary human neurons using the fluorescent inhibitor of Casp6 (FLICA) (FAM-VEID-fmk, ImmunoChemistry, Bloomington, MN, USA) following the manufacturer’s protocol. Briefly, FLICA reagent and Hoechst 33342 were added to a black clear bottom 96-well plate containing 100,000 of the treated-primary human neurons for 2 h at 37 °C in 5% CO_2_. The cells were rinsed twice with wash buffer and fresh media was added to the cells. The fluorescence of FAM-VEID-fmk was measured at 490 nm excitation and 520 nm emission with bandwidth reduced to 8 nm in the Synergy H4 plate reader (BioTek). The Hoechst signal was measured by excitation at 360 nm and emission at 485 nm.

### IC_50_ determination of NWL inhibitors on recombinant proteins or in cells

The half-maximal inhibitory concentrations (IC_50_) for NWL inhibitors (Patent publication # WO/2009/140765, WO/2010/133000, and WO/2012/140500) was determined by incubating 0 to 20 μM NWL-117 and NWL-154 with 20 nM of active site-titrated Casp6 in SB at room temperature for 5 minutes. Then, 10 μM Ac-VEID-AFC was added and fluorescence measured for 20 min at 37 °C as described above. New World Laboratories performed the IC_50_ determination for NWL inhibitors dissolved in dimethyl sulfoxide (DMSO) against recombinant Casp1-10 using the Caspase Inhibitor Drug Screening Kits (BioVision, San Francisco, CA) following the manufacturer’s instructions and the preferred substrates for the caspases (Caspase-1: WAD-AFC, Caspase-2: VDVAD-AFC, Caspase-3: DEVD-AFC, Caspase-4: LEVD-AFC, Caspase-5: WEHD-AFC, Casp6:VEID-AFC, Caspase-7: DEVD-AFC, Caspase-8: IETD-AFC, Caspase-9: LEHD-AFC, Caspase-10: AEVD-AFC). In transfected HCT116 cells, 0 to 100 μM NWL inhibitors were added in the culture media and left on the cells for 2 h. Cells were washed once with 1 mL ice-cold PBS, incubated on ice for 5 min with 200 μL cell lysis buffer (CLB) (50 mM HEPES, 0.1% CHAPS, 0.1 mM EDTA), and gently scraped off. Protein concentrations were determined by Bradford assay (BioRad, Mississauga, ON, CA) by measuring the absorbance at 595 nm using the BioTek Synergy H4 plate reader. Caspase-6 activity was measured in 40–60 μg total protein as described above. The IC_50_ for recombinant and cellular caspases were determined using GraphPad Prism 5.0 (La Jolla, CA, USA) using a log (inhibitor) – response curve with a Hill slope of −1.

### Microscopy analyses

*Immunofluorescence on human neuron cultures*: Primary human neurons were pre-treated 2 h and serum-deprived in the presence of 100 μM NWL-117, 100 μM NWL-154, or 5 μM Z-VEID-fmk (Biomol, Plymouth meeting, PA, USA) for 24 h. Following treatment, human neurons were washed once with warm PBS, fixed for 20 min at room temperature with 4% paraformaldehyde (Sigma, Oakville, ON, CA)/4% sucrose (BioRad, Mississauga, ON, CA) for TubΔCasp6 or 2% formaldehyde (Thermo Fisher Scientific, Waltham, MA, USA)/0.2% glutaraldehyde (Sigma, Oakville, ON, CA) for pBudEGFP or pBudEGFP/APP^WT^-transfected neurons [50], incubated in permeabilization buffer (0.1% Triton X-100, 0.1 sodium citrate) for 1 min on ice, washed with PBS, blocked for 20 min at room temperature with 10% goat serum (Sigma, Oakville, ON, CA), and incubated with primary antibodies diluted in 10% goat serum in PBS overnight at 4 °C in a humid chamber. The glass coverslips were washed with PBS, and incubated with goat anti-rabbit secondary antibody coupled to Alexa 488 (Molecular Probes, Eugene, OR, USA) or Cy3 (GE Healthcare Life Sciences, Baie D’Urfe, QC, CA) and Hoechst 33342 (ImmunoChemistry, Bloomington, MN, USA) at 1 μg/mL for 2 h at room temperature. The coverslips were washed with PBS and rinsed in Milli-Q water before mounting in fluoromount (Dako, Burlington, ON, CA). Images were acquired by fluorescence microscopy and quantified using the ImageJ software (NIH, Bethesda, MD, USA) for TubΔCasp6 and manually counted for transfected EGFP(+)-neurons. *Time lapse-imaging by live fluorescence microscopy*: Primary human neurons were transfected with gold beads coated with pBudEGFP or pBudEGFP/APP^WT^ using a Helios Gene gun (BioRad, Mississauga, ON, CA) [12]. Briefly, cells were pre-treated with 100 μM NWL inhibitors or 5 μM Z-VEID-fmk for 2 h. The media was removed and the neurons were shot at 100 psi. The media was quickly replaced with the inhibitors present. The plasmid was expressed for 16 h before setting up the fluorescence microscope (Nikon Eclipse Ti) to acquire 20 images per condition every hour for 72 h at 37 °C with 5% CO_2_. The images were analyzed by counting the total number of neurons (50–100 neurons per condition per independent experiment), and the number of beaded, swollen soma, and healthy neurons. In addition, the time at which cells beaded was noted. *Phase contrast microscopy:* HCT116 cells, plated at a density of 1×10^5^ cells/well and human neurons, plated at a density of 6×10^6^ cells/well on poly-L-lysine were treated with PBS, 100 μM NWL-117, 100 μM NWL-154, or 2 μM staurosporine (Biomol, Plymouth meeting, PA, USA) for 24 h. Images were acquired with the Nikon Eclipse Ti microscope and the NIS-Elements (Version 3.10) software.

### Cellular toxicity assays

*MTT assay*: HCT116 cells or primary human neurons were seeded in 96-well plates at a density of 1x10^4^ and 1x10^5^ cells per well, respectively. The next day, cells and neurons were treated with vehicle (PBS), or 20, 50, or 100 μM of NWL-117, NWL-154, or 2 μM staurosporine for 24 or 48 h. The media was replaced with 0.5 μg/ml MTT (3-(4,5-dimethylthiazol-2-yl)-2,5-diphenyltetrazolium bromide) (Sigma-Aldrich, Oakville, ON, CA) and the cells were incubated for 4 h at 37 °C in 5% CO_2_. The media was removed before dissolving the formazan crystals in 100 μL DMSO while shaking for 30 min. Once dissolved, the absorbance of each sample was measured at 560 nm and 670 nm using the Synergy H4 plate reader from BioTek (Winooski, VT, USA). *LDH Assay*: HCT116 cells were seeded in a 6-well plate at a density of 1×10^5^ cells/well and treated the following day with 100 μM NWL-117 or −154 or equal volumes of vehicle (PBS) for 24 h. As a positive control, some cells were lysed for 2 h in 0.9% Triton X-100 (BioShop Canada Inc, Burlington, Ontario, CA). Media was collected and stored at −20 °C or assayed right away using the Cytotox 96 kit (Promega, Madison, WI, USA) following the manufacturer’s protocol. Hydrochloric acid (1 N) was added for 10 min to stop the reaction, which was read at 490 nm (signal) and 520 nm (background) using the Synergy H4 plate reader from BioTek. Media without cells were also used to correct the absorbance. *SubG1 population analysis*: HCT116 cells were seeded in a 6-well plate at a density of 1x10^5^ cells/well and treated the following day with 100 μM NWL-117, 100 μM NWL-154, equal volumes of vehicle (PBS), or 2 μM staurosporine for 24 h. The media was recovered and the cells were trypsinized in 0.25% Trypsin-EDTA (Thermo Fisher Scientific, Waltham, MA, USA). Both the media and the cells were combined and centrifuged for 5 min at 4 °C and washed with cold PBS-EDTA (5 mM). Cells were resuspended in 1 mL cold PBS-EDTA (5 mM), fixed by the dropwise addition of 3 mL of ice cold 100% ethanol and stored at −20 °C overnight. After centrifugation, the ethanol was removed and the cells were washed with cold PBS-EDTA (5 mM). Then, 1 mL of staining solution was added (5 mM PBS-EDTA, 50 μg/mL propidium iodide, 20 μg/mL RNAse A (Sigma-Aldrich, Oakville, ON, CA)). The samples were analyzed by flow cytometry using the FACS Calibur II instrument (BD Biosciences, Mississauga, ON, Canada). The data were interpreted using the cell cycle analysis tool in FlowJo Version 10.0, which determined the DNA content in cells based on propidium iodide intensity (Ashland, OR, USA).

### Western blot analyses

Protein extracts from HCT116 cells and human primary neurons were subjected to western blotting analyses. The 10630 (1:10 000) and GN60622 (1:10 000) neoepitope antibodies against the p20 subunit of active Casp6 (Casp6p20) and α-tubulin cleaved by Casp6 (TubΔCasp6) were generated previously in our laboratory [4, 10, 51]. The β-actin clone AC-15 (1:5 000, Sigma-Aldrich, Oakville, ON, CA), Casp6 (1:1 000), Synapsin (1:5 000), full-length α-tubulin (1:1 000, Cell Signalling Technology Inc., Danvers, MA, USA), GFAP (1:3 000, Dako, Burlington, ON, CA), synaptophysin (1:5 000, Sigma, Oakville, ON, CA), and PSD95 clone K28143 (1:5 000, UC Davis/NIH NeuroMab Facility) were purchased. All antibodies were diluted in 5% non-fat dry milk. Secondary anti-mouse (1:5 000, GE Healthcare Life Sciences, Baie D’Urfe, QC, CA) and anti-rabbit antibodies (1:5 000, Dako, Burlington, ON, CA) conjugated to horseradish peroxidase were used to detect immunoreactive proteins using ECL prime western blotting detection reagent (GE Healthcare Life Sciences, Baie D’Urfe, QC, CA) and Kodak BioMax MR film (Kodak, Rochester, NY, USA). Secondary anti-mouse conjugated to alkaline phosphatase (Jackson Immunoresearch Laboratories Inc., West Grove, PA) was developed with nitro-blue tetrazolium (Thermo Fisher Scientific, Waltham, MA, USA) and 5-bromo-4-chloro-3-indolylphosphate (Thermo Fisher Scientific, Waltham, MA, USA) for chromogenic detection of proteins. The western blots were scanned with an HP scanner and the images were not manipulated except to adjust the brightness/contrast and this was done simultaneously to the entire blot. Quantification was performed with the ImageJ software (NIH, Bethesda, MD, USA).

### NWL caspase inhibitors and Casp6 transgenic mice

Casp6 transgenic mouse model: All animal procedures followed the Canadian Council on Animal Care guidelines and were approved by the McGill Animal care committees. Sixteen to 20 month old C57BL/6 J mice were bred and aged in the pathogen-free Goodman Cancer Research Centre Mouse Transgenic Facility at McGill University. Mice were housed in a temperature-controlled room at 22 °C and were kept on a 12 h light/dark cycle. Food and water were available at libitum. Casp6 overexpressing mice (KI/Cre) express Casp6p20p10 under the CAG promoter (CMV immediate early enchancer/chicken β-actin promoter fusion) in the CA1 pyramidal cell layer of the hippocampus under the control of calmodulin kinase IIa (CAMKIIa)-regulated Cre expression [9]. No obvious toxicity was observed after a one month treatment of NWL-117 on mice (Additional file 1).

### NWL treatments

Only males were used and littermates from each genotype (wild type (WT)/WT, WT/Cre, & knock-in (KI/)Cre) were tested together. The experimenter was blind to genotype and treatment groups. Mice were administered 20 mg/kg NWL-117 or physiological saline (0.9% NaCl) by intraperitoneal injections two times 48 h apart. Injection volumes did not exceed 150 μL of 10 mg/mL NWL-117 prepared in physiological saline. *Blood brain barrier permeability of NWL-117 caspase inhibitor*: Briefly, 18–22 month old Casp6 mice were anesthetised with isoflurane, warmed with a heating blanket, and their physiological vitals (heart rate, body temperature, and respiration) monitored. The skin over the mouse’s neck was shaved, xylocaine applied, and an incision was made along the midline. Under a surgical microscope, the right carotid artery was gently separated from surrounding tissue. Two suture threads were placed at the proximal and distal ends of the carotid, and a small incision was made along the carotid wall. A micro-catheter (attached to a 1 ml syringe controlled by a micropump) was inserted and pushed to the entrance of the internal carotid artery. The catheter was secured in place using suture thread. NWL-117 (20 mg/kg) was infused via the micro-catheter using a pump set at 50 μL/min (total volume between 64 μL and 130 μL). After 5 min, blood was collected by intra-cardiac puncture, the mouse was perfused through the heart with ice cold saline for 2–3 min. The brain was removed and hippocampi were dissected and frozen on dry ice and stored. Samples were sent to the Biopharmacy platform at the University of Montreal (Quebec, Canada) for liquid chromatography and tandem mass spectrometry analysis. The integrity of the blood-brain barrier was confirmed by injecting 3% Evan’s blue solution in 20 month old Casp6 mice. *Mouse cognitive analysis by novel object recognition*: Mice were handled during 5 min for one week prior to behavioral tests. Novel object recognition (NOR) task was administered in three phases: habituation, familiarization (pre-exposure), and test phase. For habituation, mice were placed in the NOR box (80 cm x 80 cm, Stoelting Co, Wood Dale, IL, USA) for 5 min. After 24 h, the pre-exposure phase was initiated by allowing the animals to explore two identical objects inside the NOR box. Then, following a 2 h gap, mice were re-introduced to the NOR box which now contained a familiar and a novel object. The position of the novel object was counterbalanced between animals to avoid any bias related to a preference in the location of the new object and the use of potential confounding spatial cues. The objects were located in the middle of the NW and SE quadrants of the box, equidistantly from the box corners and from each other. The mice were placed in the middle of the SW quadrant. Washing the box and objects with 70% ethanol eliminated odour cues. The number of times touching each object was manually recorded, while the total distance, percent time moving, and number of entries into virtual cells were recorded using the HVS 2100 automated video tracking system (HVS Image, Buckingham, UK). Animals whose exploration was considered insufficient to allow recognition (<10 s per object) during the familiarization phase were excluded from analysis. Different object sets were used in the pre- and post-tests. *Immunohistochemistry on mouse brain slices*: Following behavioural analysis, animals were anaesthetized under isoflurane and perfused intracardially with ice cold saline for 7 min and 4% paraformaldehyde for 20 min. Mice brains were removed and stored in 10% neutral-buffered formalin (Thermo Fischer Scientific, Waltham, MA, USA) for 24 h then dehydrated in 70% ethanol for 24 h or less. Brains were embedded in paraffin and cut using a vibratome at the histology platform of the Institute for Research on Immunology and Cancer (U Montreal). Slides containing 4 μm thick sections of the anterior hippocampus were deparaffinized in xylene (Thermo Fisher Scientific, Waltham, MA, USA), and rehydrated before demasking in antigen retrieval buffer (10 mM Tris, 1 mM EDTA, pH 9; or 10 mM tri-sodium citrate, pH 6 for synaptophysin) for 20 min at 97 °C in the Pascal Dako Cytomation (Dako, Burlington, ON, CA). The immunostaining procedure was automated using the Dako Autostainer Plus slide processor and the EnVision Flex system (Dako, Burlington, ON, CA). Slides were treated with peroxidase for 5 min, then rinsed, blocked with Serum-Free Protein Block (Dako, Burlington, ON, CA) for 30 min, and incubated with either Iba1 (1: 2 000, Wako, Richmond, VA, USA), TubΔCasp6 (1: 5 000), or synaptophysin (1: 8 000, Sigma, Oakville, ON, CA) antibodies diluted in EnVision Flex Antibody Diluent (Dako, Burlington, ON, CA) for 30 min. After rinsing, the mouse brain slices were incubated with secondary rabbit-horseradish peroxidase antibody (Dako, Burlington, ON, CA) for 30 min and diaminobenzidine (Dako, Burlington, ON, CA) for 10 min before counterstaining with hematoxylin (Dako, Burlington, ON, CA). Slides were scanned using the MIRAX SCAN (Zeiss, Oberkochen, Germany) and analyzed using the ImageJ software (NIH, Bethesda, MD, USA) by measuring the area of positive immunoreactivity over the total area in square microns.

### Statistical analysis

Statistical analysis of data was performed using Graphpad Prism 5.0 (La Jolla, CA, USA). The analyses were done with ANOVA followed by post-hoc analyses as indicated for each test in the figure legends. Alternatively, a student *t*-test was done to compare between two samples as indicated in figure legends. Significance was set at *p* < 0.05 for all experiments.

## Results

### NWL-117 and −154 are potent peptide-based vinyl methyl sulfone inhibitors of recombinant active Casp6

**Fig. 1:**
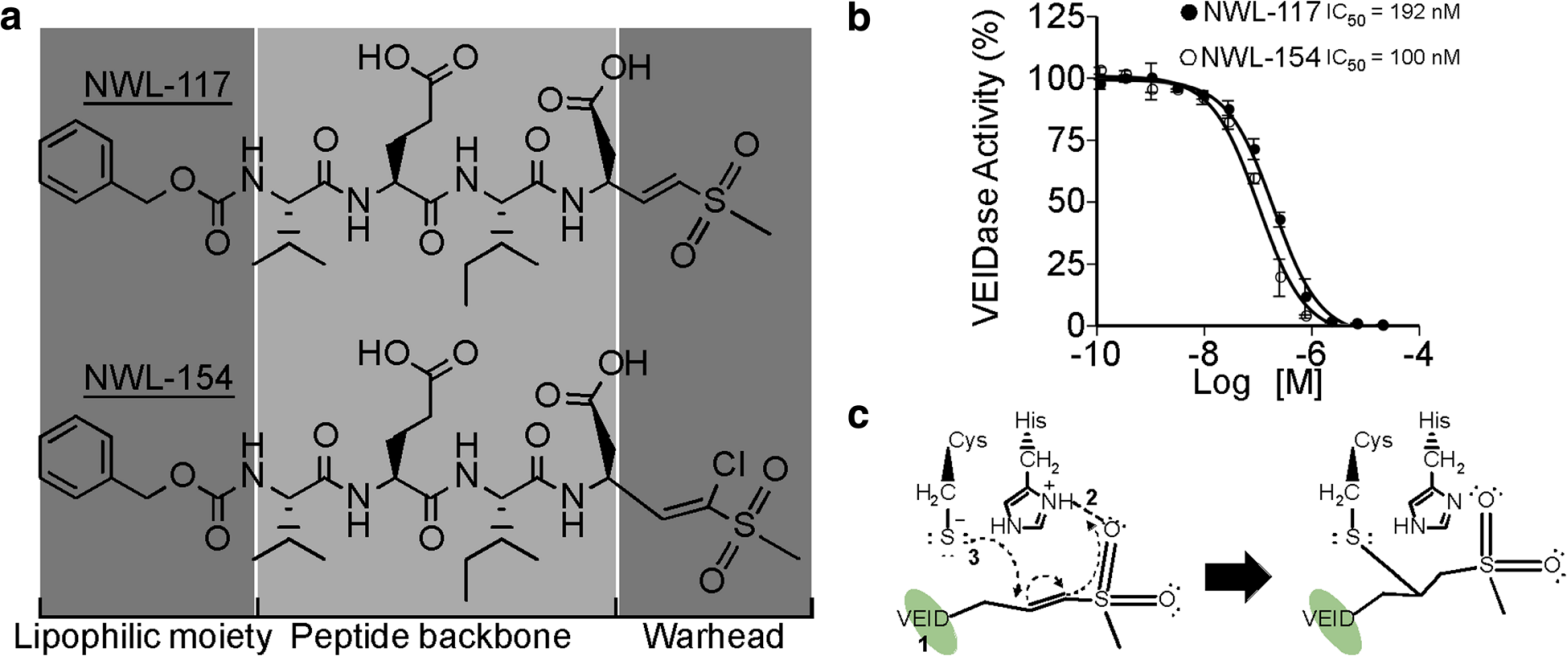
NWL inhibitors are irreversible peptide vinyl sulfone inhibitors of Casp6. **a** Chemical structures of NWL-117 and NWL-154 highlighting the lipophilic moiety, the peptide backbone, and the chemical warhead. **b** Dose-response curve for NWL-117 (*closed circle*, IC_50_ = 192 nM) or NWL-154 (*open circle*, IC_50_ = 100 nM) against 20 nM recombinant active site-titrated Casp6. Data represent the mean ± S.D. for three independent experiments. **c** Schematic representation of the mechanism for covalent linkage of vinyl sulfone warheads to the catalytic cysteine of Casp6. 1 Peptide (VEID) binds to the substrate-binding pocket. 2 This interaction allows the sulfone moiety to form a hydrogen bond with the protonated imidazole ring of histidine. 3 The sulfur from the catalytic cysteine performs a nucleophilic attack on the β-carbon of the vinyl group, which triggers a movement of electrons leading to the protonation of the α-carbon. The result is a covalent link between the vinyl sulfone inhibitor and Casp6

NWL-117 and NWL-154 are peptide-based inhibitors flanked by a lipophilic moiety and a vinyl methyl sulfone chemical warhead (Fig. 1a). NWL-117 and −154 showed a dose-dependent Casp6 inhibition with half-maximal inhibitory concentrations (IC_50_) of 192 nM and 100 nM, respectively (Fig. 1b). The peptide backbone of NWL inhibitors binds to the active site of Casp6, which allows the vinyl sulfone warhead to hydrogen bond with the protonated imidazole ring of histidine, while the catalytic cysteine attacks the β-carbon of the vinyl group (Fig. 1c). The reaction is irreversibly stabilized under physiological conditions by histidine de-protonation by the α-carbon of the vinyl group [43]. Thus, peptide-based vinyl sulfones are potent, irreversible, and competitive Casp6 inhibitors.

### NWL inhibitors inhibit recombinant initiator caspases and Casp6 at sub-micromolar concentrations

NWL inhibitor specificity was tested on recombinant Casp1 to Casp10 (Table 1). NWL-117 inhibited effector Casp6, but not Casp3 or −7. NWL-117 inhibited initiator Casp8, Casp9, and Casp10, and inflammatory Casp4 but not Casp1 and Casp5. NWL-154 inhibited most strongly Casp6, Casp8 and Casp10, but not Casp3, Casp7, Casp9, Casp1, Casp4, or Casp5. Neither compounds showed inhibitory activity on Casp2. Casp6 IC_50_ in Fig. 1b differs slightly from these results because the recombinant Casp1-10 were not active site titrated as done for Casp6 purified in-house. These results indicate that NWL-117 and NWL-154 are strong Casp6 inhibitors although their selectivity requires improvement.

**Table 1:** Half-maximal inhibitory concentrations (IC_50_)^a^ of NWL-117 and NWL-154 against recombinant Caspase-1 to −10

Cpd No.	MW^b^	Caspase-									
		1	2	3	4	5	6	7	8	9	10
NWL-117	668	2.7	>100	6.96	0.38	19.54	0.60	>100	0.66	0.79	0.13
NWL-154	703	3.85	>100	1.34	1.80	4.19	0.23	>100	0.53	6.40	0.19

^a^IC_50_ values are measured in μM

^b^Molecular weight in g/mol

### NWL-117 and −154 are non-toxic and potent inhibitors of Casp6 activity in Casp6-transfected HCT116 cells

**Fig. 2:**
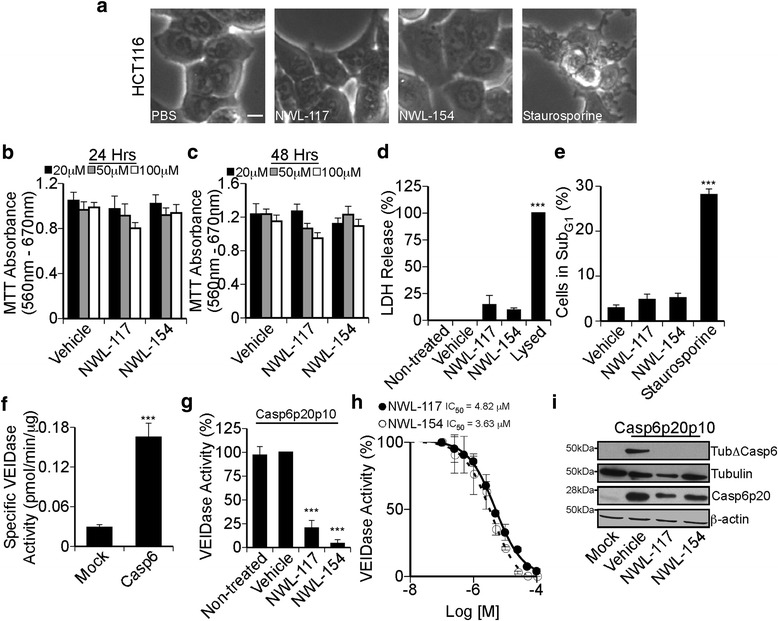
Non-toxic concentrations of NWL-117 and −154 inhibit Casp6 activity in HCT116 cells. **a** Phase contrast microscope images of HCT116 cells treated with phosphate-buffered saline, 100 μM NWL inhibitors, or 2 μM staurosporine for 24 h. Scale bar represents 10 μm. **b** & **c** MTT absorbance following treatment with PBS, NWL-117, or NWL-154 at 20, 50, or 100 μM for 24 h (**b**) or 48 h (**c**). No statistical differences were found by two-way ANOVA with Bonferroni post-tests. **d** Lactate dehydrogenase activity in untreated cells, or treated with PBS, 100 μM NWL inhibitors, or lysed with 0.9% Triton X-100 for 24 h. **e** Quantification of the sub-G_1_ population following cell cycle analysis in PBS, 100 μM NWL inhibitors, or 2 μM staurosporine treatment for 24 h. **f** VEIDase activity in pCep4β-transfected (mock) or pCep4β-Casp6p20p10 transfected HCT116 cells. Data represent the mean ± SEM of five independent experiments. Statistical analysis was performed using an unpaired two-tailed *t*-test (*** *p* < 0.01). **g** Casp6 VEIDase activity in cellular extracts from pCep4β-Casp6p20p10-transfected HCT116 cells treated with PBS, NWL-117 or NWL-154 at 100 μM for 2 h. **h** Dose-response curve for NWL-117 (*closed circle*, IC_50_ = 4.82 μM) and NWL-154 (*open circle*, IC_50_ = 3.63 μM) in pCep4β-Casp6p20p10-transfected HCT116 cells treated for 2 h. **i** Western blot analysis of samples from panel f and g for α-tubulin-cleaved by Casp6 (TubΔCasp6), α-tubulin (Tubulin), active Casp6 p20 subunit (Casp6p20), and β-actin. Casp6 expression was allowed for 24 h before treatment with inhibitors in all transfection experiments. For panels b-g, data represent the mean of three independent experiments ± SEM and were analyzed by one-way ANOVA (*p* < 0.0001) with post hoc Dunnett’s multiple comparison test comparing to vehicle-treated (***denotes *p* < 0.001) unless specified otherwise

To address NWL inhibitor efficacy in a cellular context, human colon carcinoma HCT116 cells were treated with 100 μM NWL-117, NWL-154 or staurosporine as a cell death control for 24 h (Fig. 2a). NWL inhibitor-treated cells looked morphologically normal. The mitochondrial reductive potential of cells treated with 20, 50, or 100 μM NWL-117 or −154 for 24 (Fig. 2b) or 48 h (Fig. 2c) was comparable to vehicle-treated cells. NWL-treated cells did not release lactase dehydrogenase (LDH) (Fig. 2d) nor did the cells show increased sub-G1 levels of DNA (Fig. 2e), excluding necrosis and apoptosis, respectively. Therefore, unlike staurosporine, NWL inhibitors are not toxic to HCT116 cells at the tested concentrations.

Casp6 overexpression in HCT116 increases Casp6 activity fivefold (Fig. 2f), and 100 μM NWL-117 or −154 treatments for 2 h decreased Casp6 activity by 80% (IC_50_ 4.82 μM; r^2^ = 0.94) and 96% (IC_50_ 3.63 μM; r^2^ = 0.91), respectively (Fig. 2g-h). Functional inhibition of Casp6 activity was confirmed by a substantial decrease in TubΔCasp6 and a modest reduction of active Casp6 p20 subunit in cells treated with 100 μM NWL inhibitors (Fig. 2i). These findings suggest a functional inhibition of Casp6 by NWL inhibitors within HCT116 cells.

### NWL inhibitors block Casp6 activity within minutes in Casp6-transfected HCT116 cells

**Fig. 3:**
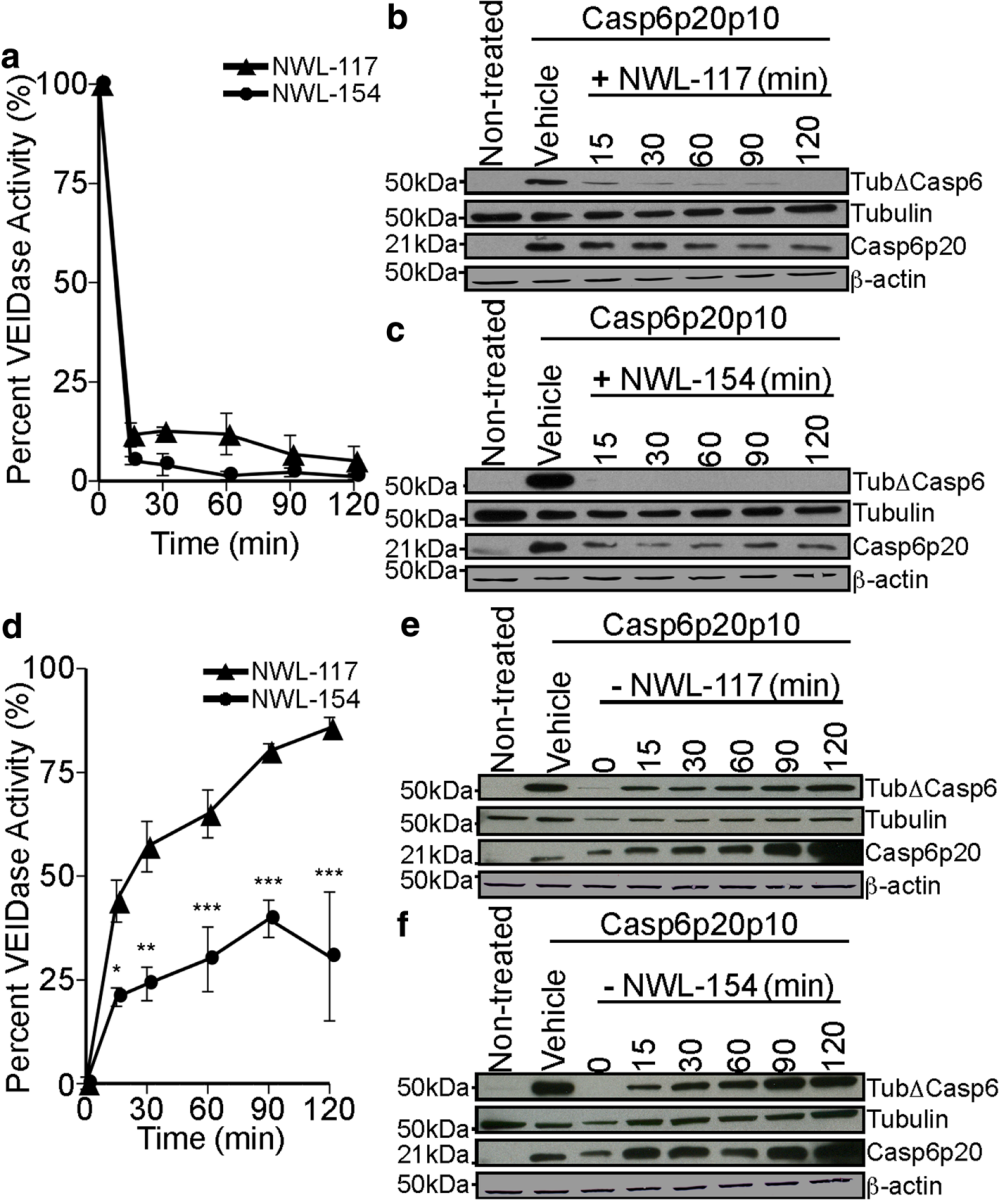
NWL-117 and −154 rapidly inhibit Casp6 activity in HCT116 cells. **a** Percent VEIDase activity from cellular protein extracts of Casp6-transfected HCT116 cells treated with either NWL-117 (*closed triangle*) or −154 (*closed circle*) at 100 μM for 0, 15, 30, 60, 90, or 120 min. No statistical significant differences were obtained between NWL-117 and NWL-154. **b** & **c** Western blot analysis of samples from panel (**a**) of NWL-117 (**b**) and NWL-154 (**c**) for α-tubulin-cleaved by Casp6 (TubΔCasp6), α-tubulin (Tubulin), active Casp6 p20 subunit (Casp6p20), and β-actin. **d** Percent VEIDase activity from cellular extracts after 2 h of treatment with 100 μM NWL-117 (*closed triangle*) or −154 (*closed circle*) in Casp6-transfected HCT116 cells followed by the removal of the inhibitors for 0, 15, 30, 60, 90, or 120 min. **e** & **f** Western blot analysis of samples from panel (**d**) of NWL-117 (**e**) and NWL-154 (**f**) for α-tubulin-cleaved by Casp6 (TubΔCasp6), α-tubulin (Tubulin), active Casp6 p20 subunit (Casp6p20), and β-actin. For panels (**a**) & (**d**), data represent the mean ± SEM of three independent experiments. Statistical analysis was performed by two-way ANOVA (((compound (*p* = 0.0010), time (*p* < 0.0001), interaction (*p* = 0.4546)); (compound (*p* < 0.0001), time (*p* < 0.0001), interaction (*p* = 0.0054))), respectively, with Bonferroni post-tests (*p < 0.05, **p < 0.01, ***p < 0.001)

To assess the kinetics of NWL-117 and −154-mediated Casp6 inhibition, Casp6-expressing HCT116 cells were treated with 100 μM NWL-117 or NWL-154 for 15 to 120 min after 24 h of transfection. Within 15 min of treatment, Casp6 VEIDase activity decreased significantly by 88% with NWL-117 and 95% with NWL-154 and the inhibition continued for 2 (Fig. 3a) and 48 h (Additional file 2: Figure S1). TubΔCasp6 and Casp6p20 levels were decreased within 15 min of treatment (Fig. 3b, c).

To assess Casp6 activity recovery, transfected cells were treated with 100 μM NWL-117 or NWL-154 for 2 h and subsequently replaced with fresh media. Casp6 activity rose by 43% ± 5.0 within the first 15 min of NWL-117 removal and recovered 85% ± 2.5 of the initial activity after 2 h (Fig. 3d). In contrast, NWL-154 withdrawal increased by 21% ± 2.2 within 15 min, reaching 30% ± 15 after 2 h. Western blot analysis showed that the levels of TubΔCasp6 and Casp6p20 (Fig. 3e–f) were consistent with the VEIDase activity restoration (Fig. 3d). Together, these results indicate that the NWL inhibitors rapidly inhibit Casp6 activity and that this inhibition can be rapidly washed out in transfected HCT116 cells.

### NWL inhibitors are non-toxic and prevent Casp6-dependent neuritic degeneration in APP^WT^-transfected human CNS neurons

**Fig. 4:**
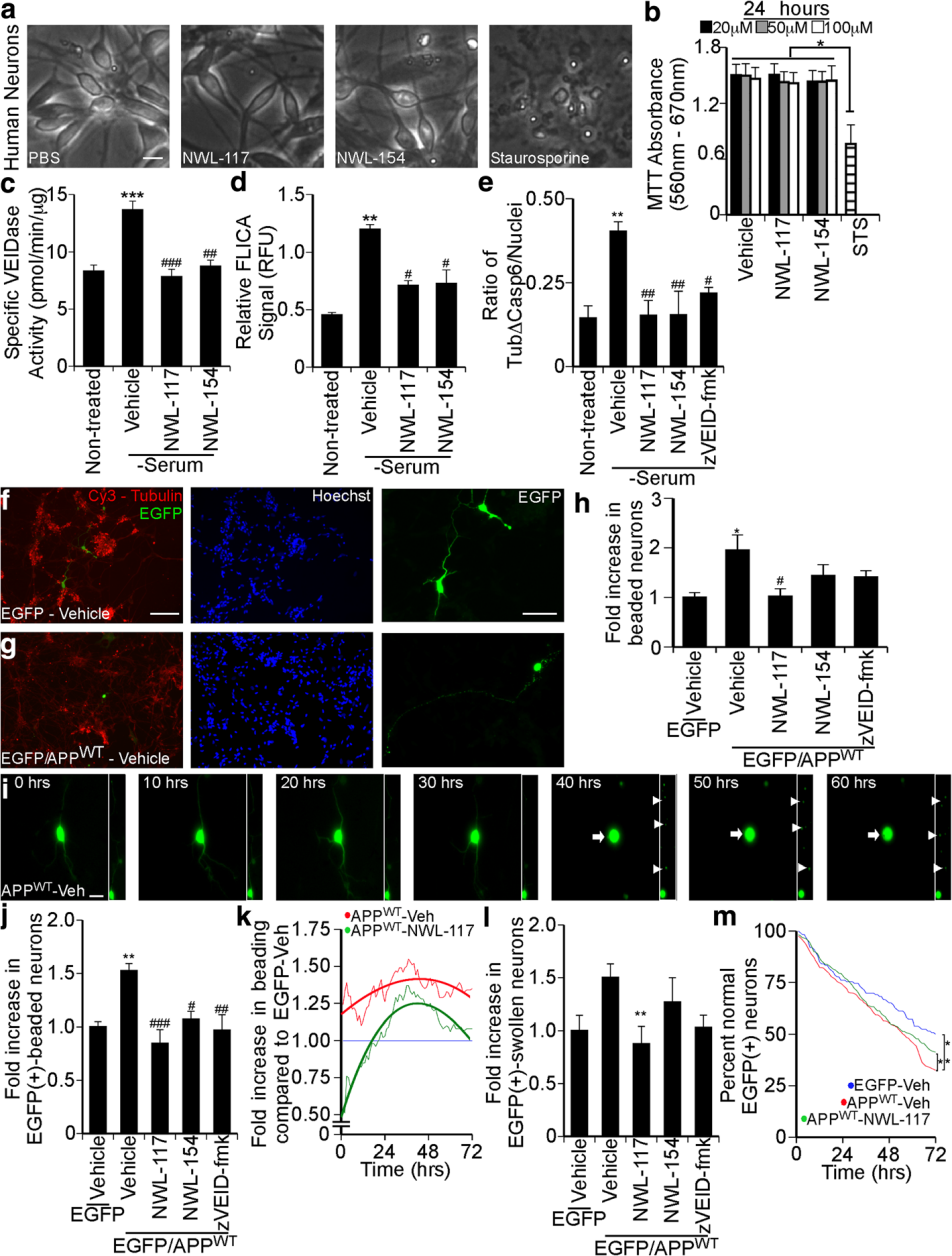
Non-toxic concentrations of NWL-117 and −154 inhibit Casp6 activity in primary human neurons. **a** Micrographs of primary human neurons treated with PBS vehicle, 100 μM NWL inhibitors, or 2 μM staurosporine for 24 h. Scale bar represents 10 μm. **b** MTT absorbance following treatment with PBS, NWL-117, or NWL-154 at 20, 50, or 100 μM or 2 μM staurosporine for 24 h (*n* = 4, one-way ANOVA (*p* = 0.0169), Tukey’s multiple comparison test (*p < 0.05)). **c** VEIDase activity in neuronal extracts following treatment with PBS, 100 μM NWL-117 (*n* = 5), or 154 (*n* = 2) for 2 h (one-way ANOVA (*p* < 0.0001)). **d** Casp6 FLICA assay (one-way ANOVA (*p* = 0.0041)). **e** Quantification of the number of TubΔCasp6 beads/nuclei in Additional file 1: Figure S2b (one-way ANOVA (*p* = 0.0095)). **f**–**h** Fluorescence micrographs following transfections with pBudEGFP (**f**) or pBudEGFP/APP^WT^ (**g**) stained for α-tubulin (Cy3), Hoechst, and quantified (**h**) (one-way ANOVA (*p* = 0.0385)). Scale bar represents 100 μm for merge and Hoechst panels, and 50 μm for EGFP panel. **i** Live-imaging fluorescence micrographs from 0 to 60 h of a human neuron transfected with pBudEGFP/APP^WT^ and pre-treated with vehicle. The inset highlights the neurite extending upward. Arrowheads indicate agglomerates of EGFP protein within the axonal membrane while arrows mark a rounded cell body. Scale bar represents 10 μm. **j**–**m** Quantification of panel (**i**) for overall fold increased beaded neurites (**j**), fold increased beaded neurons at specific times (**k**), overall fold increase swollen neuronal soma (**l**), or normal EGFP positive neurons (**m**). For panels (**c**–**e**, **h**), and (**j**–**k**) data represent the mean ± SEM (*n* ≥ 3), one-way ANOVA (*p* = 0.0005 for j, *p* =0.0264 for k, and post hoc tests were performed with Dunnett’s multiple comparison test (*compares to serum (+) or EGFP-vehicle: *p < 0.05, **p < 0.01, ***p < 0.001; # compares to serum (−) with vehicle or to EGFP/APP^WT^-vehicle: # *p* < 0.05, ## *p* < 0.01, ### *p* < 0.001) unless stated otherwise. For panel (**m**), log-rank Mantel-Cox test was performed to compare between curves

Human primary neurons are primary targets for caspase inhibitors in neurodegenerating brains, and therefore these were cells of choice to examine the potential toxicity and caspase inhibition by these vinyl sulfone caspase inhibitors. Treatment with 100 μM NWL-117 or NWL-154 for 24 h did not change neuronal morphology (Fig. 4a), and showed mitochondrial reductive potential comparable to vehicle-treated neurons at 24 (Fig. 4b) or 48 h (Additional file 2: Figure S2a). These results indicate that neither NWL-117 nor −154 is toxic at concentrations up to 100 μM in human neurons.

Casp6 fluorogenic (Fig. 4c) and fluorescent Casp6 inhibitor (FLICA)-measured activity (Fig. 4d) and Tub∆Casp6 (Fig. 4e) were decreased by both inhibitors in serum-deprived human neurons, thus supporting the ability of NWL-117 and −154 to inhibit intraneuronal Casp6 activity. Co-expression of APP^WT^ and enhanced green fluorescent protein (EGFP) induced neuritic beading and somatic swelling after 48 h (Fig. 4f, g), as previously observed [12]. NWL-117, NWL-154, or 5 μM Z-VEID-fmk prevented neuritic beading (Fig. 4h). To study this effect over time, pBudEGFP- (Additional file 2: Figure S3a) or pBudEGFP/APP^WT^_−_transfected neurons treated with vehicle (Fig. 4i) or NWL-117 (Additional file 2: Figure S3b) were assessed for beading by live fluorescent microscopy for a 72 h period. The percentage of beaded neurons increased significantly by 1.52 fold ± 0.07 with APP^WT^ compared to EGFP-alone (1.00 ± 0.05) and decreased to 0.84 fold ± 0.13, 1.07 fold ± 0.08, 0.97 fold ± 0.14 with NWL-117, −154, or 5 μM Z-VEID-fmk treatment, respectively (Fig. 4j). Analyses with time indicate that the NWL-117-treated neurons have less beading compared to both pBudEGFP-transfected and pBudEGFP/APP^WT^_−_transfected neurons (Fig. 4k). This protection is attenuated after 24 h because media was not replenished with the NWL-117 to avoid loosing the settings for the time-lapse microscopy. Similarly, neuronal soma rounding was inhibited by NWL-117 (Fig. 4l). Finally, a measure of neurons with homogeneously distributed EGFP as a measure of health shows that NWL-117 significantly increases neuronal survival under these conditions (Fig. 4m) Therefore, NWL-117 showed stronger neuroprotective effects than NWL-154 against neuronal beading and rounding in the APP^WT^ -transfected human neurons.

### NWL-117 penetrates the blood-brain barrier and reaches high nanomolar concentrations in mouse brains

To assess the blood-brain barrier permeability of NWL-117, 18 month old Casp6-expressing transgenic mice were injected via the carotid artery. Liquid chromatography/tandem mass spectrometry (LC/MS-MS) analyses showed hippocampal concentrations ranging from 67.9 nM to 879 nM, while plasma concentrations ranged from 3.4 μM to 48 μM, after 5 minutes of injection (Table 2). The ratio between hippocampal and plasma concentrations suggested low brain penetrance, although levels greater than the in vitro IC_50_ against Casp6 (192 nM) and some initiator caspases (Table 1) were reached. Variability was expected due to the unpredictable effects of surgery on old mice. Blood-brain barrier integrity was confirmed with Evan’s blue. These results demonstrate the ability of NWL-117 to cross the blood-brain barrier in mice.

**Table 2:** NWL-117 levels in mice hippocampi and plasma following carotid artery injections^a^

Animal #	Hippocampus	Plasma	Ratio^b^
1	193	48521	0.004
2	816	3416	0.239
3	67.9	8998	0.008
4	356	7408	0.048
5	879	25425	0.035

^a^Concentrations are reported in nM

^b^Ratio of [hippocampal]/[plasma]

### Treatment of human Casp6 knock-in mice with NWL-117 improves their performance in the novel object recognition (NOR) task

**Fig. 5:**
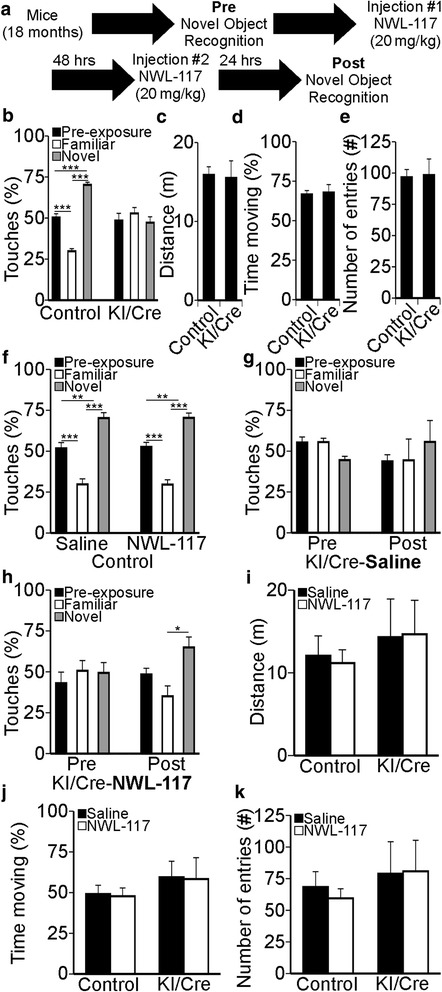
Acute NWL-117 administration reverses novel object recognition deficits in Casp6-overexpressing KI/Cre mice. **a** Experimental design for the in vivo study highlighting the novel object recognition (NOR) tests and injections. **b** Percent touches of objects during the NOR task in WT/WT and WT/Cre controls (*n* = 16), and Casp6-expressing KI/Cre (*n* = 9) mice prior to injections. Statistical analysis was performed by one-way ANOVA (*p* < 0.0001). **c** Distance traveled, (**d**) percent time moving, and (**e**) number of cell entries of control (*n* = 16) and KI/Cre (*n* = 9) mice. Statistical analysis was performed by unpaired two-tailed *t* test. No significant differences were found in C-E. **f** Percent touches of objects during the NOR task following saline (*n* = 8) or 20 mg/Kg NWL-117 (*n* = 8) injections in control mice. Statistical analysis was performed by one-way ANOVA (*p* < 0.0001). **g** Percent touches of objects during the NOR task in pre- and post-injections (*n* = 4) saline injections in KI/Cre mice. Statistical analysis was performed by repeated measures ANOVA (*p* = 0.7661). **h** Percent touches of objects during the NOR task in pre- and post- (*n* = 5) 20 mg/Kg NWL-117 injections in KI/Cre mice. Statistical analysis was performed by repeated measures ANOVA (*p* = 0.0860) with Bonferroni’s multiple comparison test (* *p* < 0.05). **i** Distance traveled, (**j**) percent time moving, and (**k**) number of cell entries of control mice injected with saline (*n* = 8) or NWL-117 (*n* = 8) and KI/Cre injected with saline (*n* = 4) or NWL-117 (*n* = 5). Statistical analysis was performed by two-way ANOVA for panels (**i**) to (**k**) and no significant differences were found. For panels (**a**–**k**), data represent the mean ± SEM and post hoc analyzes were performed using Bonferroni’s multiple comparison test (*p < 0.05, **p < 0.01, and ***p < 0.001) unless stated otherwise

Human Casp6 overexpression in the CA1 region of the hippocampus results in age-dependent episodic memory impairments measured by NOR [9]. Casp6 KI/Cre mice were tested following the experimental paradigm shown in Fig. 5a. Control mice spent more time with the novel object (70% ± 1.6), while KI/Cre mice did not (47% ± 3.6) (Fig. 5b) in the pre-test. Total path length (Fig. 5c), the percentage of time moving (Fig. 5d), and total number of entries in each part of the arena (Fig. 5e) were equivalent in control and KI/Cre mice indicating comparable locomotor and exploratory activities. Following two intraperitoneal injections, NWL-117-treated control mice performed equally to saline-treated mice (Fig. 5f). In contrast, saline-treated KI/Cre mice remained impaired (Fig. 5g), whereas NWL-117-treated mice regained normal NOR performance (Fig. 5h). Injections had no effect on the locomotor or exploratory activities (Fig. 5i–k). Hence, age-dependent Casp6-mediated deficits in NOR can be overcome by an acute treatment with NWL-117.

### Casp6 substrates, synaptic proteins, and glial inflammation markers are unchanged in mice hippocampi following NWL-117 treatment

**Fig. 6:**
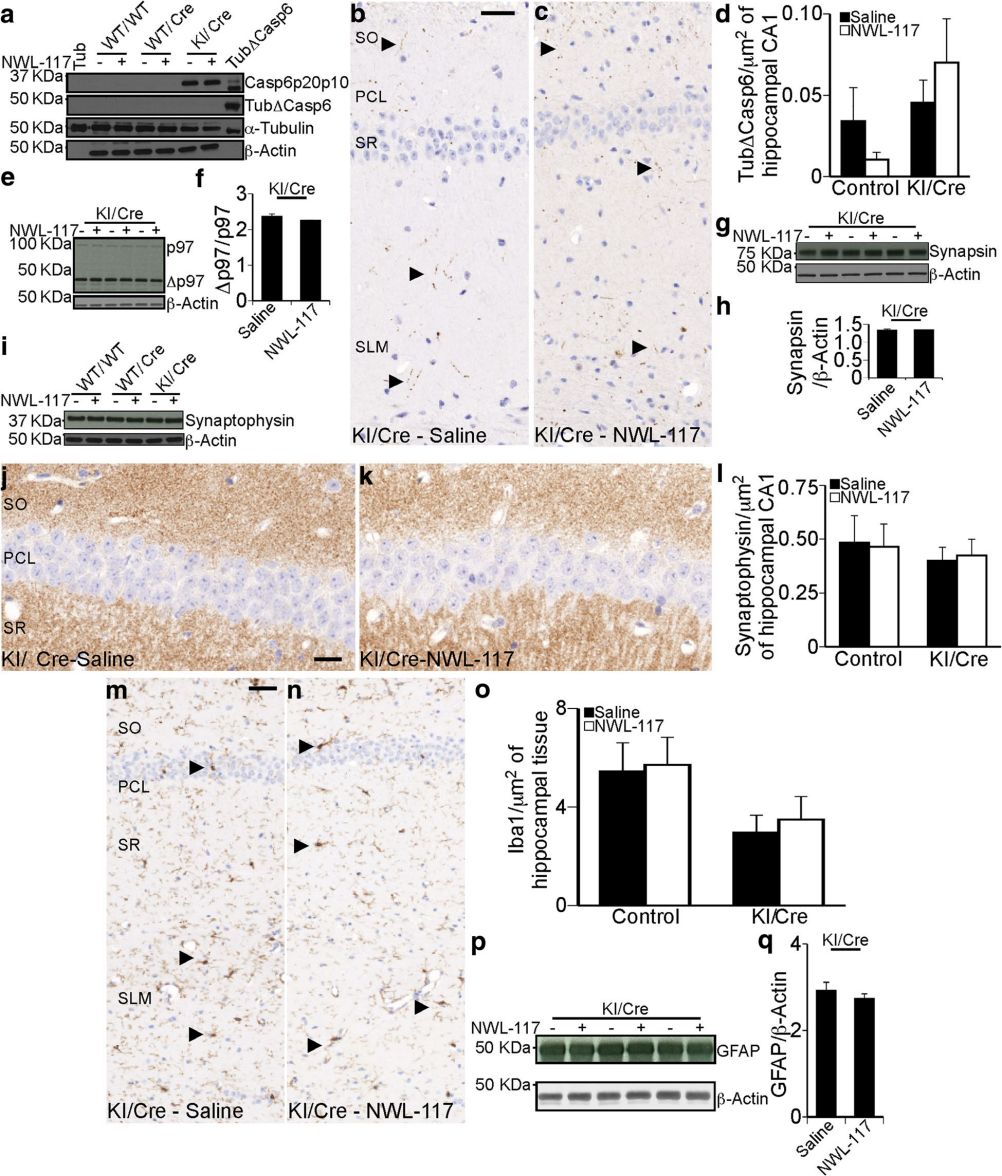
Hippocampal levels of synaptic and glial markers are unchanged with NWL-117 treatment. **a** Levels of Casp6 p20p10 (Casp6p20p10), α-tubulin-cleaved by Casp6 (TubΔCasp6), α-tubulin (Tubulin), and β-actin in hippocampal protein extracts WT/WT, WT/Cre, and KI/Cre mice treated with saline or 20 mg/Kg NWL-117. **b** & **c** Immunohistochemistry of TubΔCasp6-stained hippocampi from KI/Cre mice treated with saline (**b**) or NWL-117 (**c**). Arrowheads mark some positive immunoreactivity. **d** Quantification of positive immunostaining shown in panel (**b**) and (**c**). **e** Levels of p97 and p97-cleaved by Casp6 (p97ΔCasp6) in hippocampal protein extracts of KI/Cre mice treated with saline (*n* = 3) or NWL-117 (*n* = 3). **f** Quantification of the levels of p97ΔCasp6/p97 shown in panel (**e**). **g** & **h** Levels of synapsin in hippocampal extracts from KI/Cre mice treated with saline (*n* = 3) or NWL-117 (*n* = 3) (**g**) and quantified in (**h**). **i** Levels of Synaptophysin in hippocampal protein extracts from WT/WT, WT/Cre, and KI/Cre (Casp6 overexpressing) mice treated with saline or NWL-117. **j**–**k** Brightfield scans of KI/Cre-Saline (**j**) and NWL-117 (**k**) mice brains stained for synaptophysin by immunohistochemistry and quantification (**l**). **m** & **n** Iba1-stained hippocampi from KI/Cre mice treated with saline (**m**) or NWL-117 (**n**). Arrowheads mark some positive immunoreactivity. **o** Quantification of the area of positive immunostaining for Iba1 over the total area of the tissue. **p** Levels of GFAP in hippocampal extracts from KI/Cre mice treated with saline (*n* = 3) or NWL-117 (*n* = 3). **q** Quantification of the levels of GFAP/β-actin. For panels (**d**), (**l**), and (**o**), data represent the mean ± SEM for each group: Control-saline (*n* = 5), Control-NWL-117 (*n* = 5), KI/Cre-saline (*n* = 5), and KI/Cre-NWL-117 (*n* = 4). Statistical analysis was performed by two-way ANOVA and no significant differences were found. For panels (**f**), (**h**), and (**q**), data represent the mean ± SEM and statistical analysis was performed by unpaired two-tailed *t* test, no significant differences were found. SO: Stratum Oriens, PCL: Pyramidal Cell layer, SR: Stratum Radiatum, SLM: Stratum Lacunosum

Western blot analyses (Fig. 6a) showed that old KI/Cre mice overexpressed Casp6 but TubΔCasp6 levels remained below detection, as expected since neurons are likely degenerated. TubΔCasp6 positive immunohistochemical staining in the CA1 region (Fig. 6b, c) was slightly higher in saline KI/Cre mice compared to controls (Fig. 6d). NWL-117-treatment non-significantly decreased TubΔCasp6 levels threefold in control, but increased non-significantly in KI/Cre mice. Valosin-containing protein p97 cleaved by Casp6 (Δp97) (Fig. 6e) was not different in saline and NWL-117 treatments (Fig. 6f).

Synapsin, synaptophysin, and post-synaptic protein (PSD95) hippocampal protein levels were unchanged with NWL-117 treatment (Fig. 6g–i, Additional file 2: Figure S4). Synaptophysin immunoreactivity in the hippocampal CA1 region was slightly higher in saline-treated control than KI/Cre mice, and unchanged in NWL-117-treated mice (Fig. 6j–l).

Protein analyses of mouse hippocampi showed a non-significant reduction in microglial ionized calcium binding adapter molecule 1 (Iba1) levels in KI/Cre mice, whether treated or not with NWL-117 (Fig. 6m-o). Astroglial glial fibrillary acidic protein (GFAP) levels also did not change with NWL-117 treatment (Fig. 6p-q). Thus, analyses could not detect changes in synaptic proteins, Casp6 substrates, or inflammatory markers that account for the behavioral improvement seen in NWL-117-treated KI/Cre mice.

## Discussion

Our study demonstrates that NWL inhibitors are 1) non-toxic, but non-selective, strong Casp6 inhibitors in vitro, in colon cancer cells, and in primary CNS human neurons, 2) protective against Casp6-mediated neuritic degeneration in serum-deprived or APP^WT^ expressing human neurons_,_ 3) blood-brain barrier permeable, and 4) reversing episodic memory impairments in transgenic Casp6 mice.

There are several advantages to these vinyl sulfone inhibitors. NWL-117 and NWL-154 are potent, non-toxic, but non-selective Casp6 inhibitors. Both NWL-117 and NWL-154 inhibited 1) recombinant Casp6 activity (IC_50_ = 192 nM and 100 nM, respectively), 2) Casp6 activity in Casp6-transfected HCT116 cells (IC_50_ = 4.82 μM and 3.63 μM, respectively), and 3) Casp6 activity in serum-deprived human neurons. In contrast to Z-VEID-fmk [42], vinyl sulfone inhibitors remain intact after target engagement [43], and are not toxic to mammals [44]. Similarly, NWL vinyl sulfone inhibitors do not cause cellular toxicity measured by mitochondrial activity, cell morphology, LDH release, or sub-G1 populations, or any gross physiological or anatomical changes in vivo (supplementary document: pathology report). Furthermore, NWL inhibitors non-covalently interact with the substrate-binding pocket of Casp6 and effectively block other substrates from entering the active site. In addition, by bringing the weak vinyl sulfone electrophilic warhead, near the catalytic histidine and cysteine residues of Casp6, Casp6 enzymatic activity is irreversibly blocked. Compared to reversible inhibitors, irreversible inhibitors can achieve higher potency by completely inhibiting their target and require less frequent and lower doses resulting in higher safety profiles [52]. Therefore, the potential that NWL vinyl sulfone inhibitors could be used in humans is high. On the other hand, as with other active-site directed Casp6 inhibitors [18, 34–37, 41, 53, 54], NWL inhibitors remain non-selective for Casp6 since they inhibit many other caspases. Reducing the concentration of NWL inhibitors can increase selectivity, but significant enhancements in potency and specificity are still required before these inhibitors reach clinical trials. To overcome this limitation, the unique inactive conformation of Casp6 was targeted by others [55]. The peptide inhibitor, pep419, targets and stabilizes the tetrameric inactive form of Casp6 in a pH-dependent non-competitive manner in vitro and in cells [56]. Similarly, non-competitive ligands that stabilize the L2 loop of Casp6, which normally rearranges during activation [57], and a potent uncompetitive inhibitor targeting the caspase-substrate interface [58], in vitro*,* are effective Casp6 inhibitors. Through these innovative mechanisms, highly specific inhibitors have emerged, yet remain to be tested for toxicity and efficiency in cells and in mice. Nevertheless, active site inhibitors can be chemically modified to reach exquisite selectivity against specific caspases [59].

The selectivity of VEID is controversial but it remains the best candidate for targeting the active site of Casp6. The study by McStay et al. [60] does suggest that VEID can be cleaved by recombinant Caspase-3 or by Caspase-3 in extracts from Jurkat cells undergoing intrinsic apoptosis after the addition of cytochrome c and ATP, which activates the apoptosome pathway. However, other groups have shown that Casp6 is better at cleaving VEID than Caspase-3 [61, 62]. In fact, the Michaelis-Menten constant (K_m_) for VEID is 8-fold lower for recombinant Casp6 (30 μm) than it is for Caspase-3 (250 μm) [37]. This suggests that VEID binds the active site of Casp6 with greater affinity than that of Caspase-3. Similarly, the IC_50_ and the inhibitory constant (K_i_) of z-VEID-CHO (aldehyde) are 2-fold smaller for Casp6 than they are for Caspase-3 [58]. These data suggest that VEID is a preferred substrate of Casp6, although not specific. Apart from the peptide sequence, the other components of the small molecule also influence its selectivity. This is evident in [63] where screening of peptide acyloxymethyl ketones (AOMK) inhibitors resulted in the identification of TETD as the preferred peptide sequence for Casp6 over Caspase-3 [41]. Yet, in the same study, they found that Cy5 labeled VEID-AOMK was a better substrate for Casp6 than the TETD version. Similarly, chemical warheads can affect the selectivity of inhibitors bearing the same peptide sequence. In fact, VEID-CHO was more selective for Casp6 than −3 compared to the fluoromethyl ketone (FMK) counterpart [64]. Exosites also modulate substrate binding [65]. It is possible that exosites are responsible for limiting the cleavage of lamin A at the VEID sequence to Casp6 [62]. In our study, we find Z-VEID vinyl methyl sulfone inhibitors are 10-fold more selective against Casp6 than Caspase-3 (Table 1: IC_50_). It is possible that interactions of the lipophilic moiety or the chemical warhead with natural substrate exosites increase selectivity of the NWL inhibitors further towards Casp6.

Our results demonstrate that the NWL vinyl sulfone caspase inhibitors are non toxic to human neurons and neuroprotective against serum deprivation or APP overexpression. NWL inhibitors prevent serum-deprivation- or APP^WT^-expression induced TubΔCasp6 and neuritic degeneration in human neurons, as shown previously with Z-VEID-fmk and Casp6 dominant negative inhibitors [12]. Maintaining full-length α-tubulin is essential to stabilize microtubules [66], and intact microtubules are critical for neuronal function. Since active Casp6 or TubΔCasp6 are increased in human AD and hypoxia-induced ischemia [4, 10, 27], inhibition of Casp6 and other caspases in these conditions may help maintain neuronal function. Even if Casp6 has been implicated in axonal degeneration of NGF-dependent neurons, recent evidence suggests that Casp3 also participates in axonal degeneration [15, 19–26, 67]. In our study, the possibility that NWL inhibitors are acting on Casp3 to protect neurons was excluded because only Casp1 and Casp6 are co-activated in our cellular model [12, 68, 69], and both NWL-117 and NWL-154 are more effective against Casp6 than Casp1 and Casp3. Moreover, in AD brains, active Casp6 is detected in the absence of Casp3 [6, 70]. Thus, although studies in mouse peripheral neuron cultures implicate Casp3 to be an important regulator of axonal degeneration, the pathways involved in human CNS neurons seem to converge on Casp6. Nevertheless, given the strong inhibition of initiator caspases by the NWL vinyl sulfone caspase inhibitors, it is not possible in these experiments to conclude that the effect observed was uniquely due to Casp6. The ability of short-term treatment with NWL-117 to reverse episodic memory impairments in our mice suggests that Casp6-mediated damage is reversible in aged mice. We did not determine whether other caspases are activated downstream of Casp6 in our mouse model and it remains a possibility that the inhibition of other caspases such as Casp4, 8, 9, and 10 additionally contributed to the improvement of cognitive deficits mediated by the over-expression of Casp6. Nevertheless, the development of specific Casp6 inhibitors and assessment of target engagement will help determine whether it is Casp6 inhibition alone, a combination of Casp6 with other caspases activated as a consequence of Casp6 activation in the mice brains, or a non-caspase effect that is responsible for the behavioural improvement. Most importantly, our findings suggest that vinyl sulfone NWL caspase inhibitors are permeable to the blood-brain barrier with concentrations in the hippocampus reaching in vitro IC_50_ values. Intra-carotid injections were used as a proof of principle as it limits exposure to peripheral tissues and is the most rapid path to the brain. Rapid exposure was necessary as the LC/MS-MS method can only detect free NWL-117. Pharmacokinetic and pharmacodynamic detailed analyses should be conducted in order to determine dosing regimens for chronic administration studies. Only one other Casp6 inhibitor, ED11, was shown to be brain permeable and have a significant effect against behavioral and cognitive deficits in an Huntington’s mouse model [18]. ED11 was delivered by subcutaneous pump delivery of 4 mg/kg/day for 28 days or more. In contrast, NWL-117 reversed Casp6-induced memory deficits after only 2 intraperitoneal injections of 20 mg/kg within 72 h in the Casp6 transgenic mouse. These results support delivery of the NWL inhibitors to the brain and suggest that Casp6-mediated functional impairment can be rapidly reversed.

Compared to ED11 [18], NWL have several advantages. ED11 is a 24 amino acid peptide (GRKKRRQRRRPPQSSEIVLDGTDN) containing part of the human immunodeficiency virus (HIV) TAT-peptide and huntingtin protein sequences. The TAT-peptide confers permeability to plasma membranes and the blood-brain barrier, while the huntingtin sequence is used to target Casp6. NWL inhibitors have 1) lower molecular weight, 2) lower IC_50_ against VEID substrates, and 3) no activity enhancing effect on caspases compared to ED11. Lower molecular weight is associated with several different parameters that determine the oral bioavailability of compounds as well as their production cost [71]. In addition, large peptides have lower half-lives in the body due to extensive degradation and clearance by the liver and kidneys. This often leads to the use of parenteral routes of administration, like injections for insulin, which subsequently leads to reduced patient compliance. Thus, the development of small molecules is often preferred. In addition, although the IC_50_ against mutant huntingtin cleavage determined by fluorescence resonance energy transfer (FRET) was 12.12 nM for ED11, ED11 did not inhibit the cleavage of VEID-aminoluciferin as potently (>10 μM). Like NWL inhibitors, ED11 showed inhibition of other caspases, but the IC_50_ of ED11 against all caspases on their preferred substrates has not been determined. Therefore, it is not possible to compare the selectivity of ED11 to that of NWL. Furthermore, ED11 showed a significant enhancement of Caspase-5 activity on the FRET assay. Deregulated Caspase-5 activation could perturb inflammasome signalling [72]. No activation of caspases was observed with NWL inhibitors. Finally, ED11 is a competitive reversible inhibitor that gets cleaved by Casp6. Although it has not been measured, the affinity of cleaved ED11 could be lower than the parent compound. Thus, the efficacy of ED11 will be reduced over time. In contrast, irreversible NWL inhibitors can completely inhibit the target enzyme, require less frequent dosing, and have better safety profiles than reversible inhibitors [52]. In theory, both these caspase inhibitors are in the early phases of development and will need much improvement before being considered for clinical use.

The underlying molecular mechanism(s) involved in the restoration of cognitive function remain unclear. NWL-117 had no effect on hippocampal levels of TubΔCasp6 or p97ΔCasp6, synaptic protein expression, or glial inflammation markers. Our inability to detect changes may be a consequence of the short treatment period. Furthermore, Casp6 expression and activation is limited to the pyramidal neurons of the CA1 region of the hippocampus and this does not provide sufficient material to assess Casp6-cleaved protein substrates by western blot, especially since neurons will not all degenerate at the same time. In addition, the rapid reversal of cognitive deficits suggest that the effect is possibly mediated through neuronal plasticity which re-establishes neuronal function, therefore, different tools encompassing synaptic plasticity need to be developed to assess how NWL reverses cognitive deficits in these mice. In other mice studies, cognitive amelioration was measured in the absence of changes in synaptic protein expression or brain volume [18, 73]. Future long-term prophylactic treatment studies may enlighten us to the effects of NWL-117 in the context of age and Casp6-dependent cognitive impairment and allow proper identification of target engagement. In addition, NWL inhibitors need to be administered to different AD mouse models which display an aggravated pathological phenotype, such as high Aβ load or NFTs, to determine the efficacy of this treatment in re-establishing memory function in other models of neurodegeneration.

NWL inhibitors have advantages over current research tools as they are permeable to the blood brain barrier and are less toxic than the commercially available fluoromethylketone based tools (cell permeable inhibitors and FLICA reagents) without any compromise in selectivity. As NWL Inc. improves on their small molecules Casp6 inhibitors, experiments on non-human primates, clinical trials, and the development of radio-ligands for positron emission tomography will become reality.

## Conclusion

This study reveals the potential for vinyl sulfone caspase inhibitors to effectively inhibit Casp6 activity and promote neuronal axonal integrity. Also, our results suggest that Casp6-mediated damage can be reversed in aged brains. Much work still needs to be done to confirm target engagement, measure selectivity, potency, and blood-brain-barrier permeability in animal models. However, with the increasing number of research groups focusing on Casp6 as a therapeutic target against neurodegenerative diseases, the possibility that Casp6 inhibitors will one day reach human trials is promising. Whether Casp6 inhibitors will be sufficient as a monotherapy, or whether they will become part of a combinatorial approach with Tau, amyloid, and other emerging therapies is a question that will be answered in the years to come.

## Additional files

Additional file 1: Pathology report. Contains pharmacokinetic and toxicological analysis of CD-1 mice treated with NWL-117 for 28 days every 3 days. (PDF 3742 kb)
Additional file 2: Figures S1-S4. Contains additional information to complement Figs. 3, 4, and 6 of the manuscript. (PDF 463 kb)

## Abbreviations

Ac-VEID-AFC: Ac-Val-Glu-Ile-Asp-(7-Amino-4-trifluoromethylcouramin)
AD: Alzheimer disease
ANOVA: Analysis of variance
APPWT: Wild type amyloid precursor protein
Aβ: Amyloid-beta peptide
BSA: Bovine serum albumin
CAG: CMV immediate early enchancer/chicken β-actin promoter fusion
CAMKIIa: Calmodulin kinase IIa
Casp6: Caspase-6
Casp6p20: p20 subunit of active Casp6
CHAPS: 3-[(3-cholamidopropyl)-dimethylammonio]-2-hydroxy-1-propanesulfonic acid
CIF: Caspase inhibitory factor
CNS: Central nervous system
DMSO: Dimethyl sulfoxide
EDTA: Ethylenediaminetetraacetic acid
EGFP: Enhanced green fluorescent protein
FLICA: Fluorescent inhibitor of Casp6
Fmk: Fluoromethyl ketones
GFAP: Glial fibrillary acidic protein
HCT116: Human colon carcinoma cell line
IAP: Inhibitors of apoptosis proteins
Iba1: Ionized calcium binding adapter molecule 1
IC50: Half-maximal inhibitory concentrations
IPTG: Isopropyl β-D-1-thiogalactopyranoside
KI: Knock in
LC/MS-MS: Liquid chromatography/tandem mass spectrometry
LDH: Lactate dehydrogenase
MTT: 3-(4,5-dimethylthiazol-2-yl)-2,5-diphenyltetrazolium bromide
NFT: Neurofibrillary tangles
NGF: Nerve growth factor
NOR: Novel object recognition
NWL: New World Laboratories Inc.
PBS: Phosphate-buffered saline
PEG: Polyethylene glycol
PIPES: Piperazine-N, N-bis (2-ethanesulfonic acid)
PSD95: Post-synaptic protein
SB: Stennicke’s buffer
TauΔCasp6: Tau-cleaved by Casp6
TubΔCasp6: Tubulin cleaved by Casp6
WT: Wild type
Z-VAD-fmk: N-benzyloxycarbonyl-Val-Ala-Asp-(O-methyl)-fluoromethylketone
Z-VEID-fmk: Benzyloxycarbonyl-Val-Glu-Ile-Asp-fmk
Δp97: p97 cleaved by Casp6
