# Supplementary material for: Caspase vinyl sulfone small molecule inhibitors prevent axonal degeneration in human neurons and reverse cognitive impairment in Caspase-6-overexpressing mice
Source: Mol Neurodegener. 2017 Feb 28;12:22. doi: 10.1186/s13024-017-0166-z (PMC5329948; doi:10.1186/s13024-017-0166-z)
Supplement: Additional file 1: — Pathology report. Contains pharmacokinetic and toxicological analysis of CD-1 mice treated with NWL-117 for 28 days every 3 days. (PDF 3742 kb) [file 13024_2017_166_MOESM1_ESM.pdf]

Title: Pharmacokinetic and toxicological evaluation of novel peptide-based drugs. (NWL-117Na)

Study plan version: 1
Principal investigator: Jan-Eric Ahlfors
Research Team: Annie Salesse DEC., Richard Frenette M.Sc. Scientist, Medicinal Chemistry

Summary: We have developed novel peptide-based drugs for treating various neurological conditions as well as for promoting neuronal repair. As part of the drug development process, it is necessary to evaluate the pharmacokinetic (PK) and toxicological parameters of these drug products in healthy animals to further validate the safety and efficacy of our drug products. In this study we evaluated the toxicity associated with a dose of 50 mg/kg, by subjecting the animals to multiple doses of the drug over a period of 28 days.
Background: A 28-day repeat dose toxicity evaluation was performed on CD1 mice at MISPRO (an AAALAC approved animal facility) for our parent drug (Compound X) using 50 mg/kg/day drug concentration. We observed no significant adverse effects or toxicity in CD1 mice when the Compound X was administered intraperitoneally. Since the drugs that we will be evaluating in this study are chemical derivatives of our parent drug (slight modification in functional groups on the parent structure to improve specificity and potency of the derivative drugs), we anticipate that there will not be any significant change in the EC50 of the derivative drugs. We also hypothesize that at higher doses, our parent drug and its derivatives shouldn't have significant toxicity. This is based on our earlier findings from CD1 mice that the Compound X was rapidly metabolized and cleared, leaving no molecular signatures or metabolites that may cause deleterious effect.
Study objectives: The objective of this study was to determine the safety, toxicity, and local tissue reaction to repeated doses of NWL-117Na at 50 mg/kg IP. To evaluate if a specific compound has any local reaction, a small pilot study of 8 animals (repeat dose, 50 mg/kg injected IP) will be performed first to determine if there are local toxic or side-effects of a compound. This pilot study will also simultaneously determine if a particular compound has high toxicity by any chance. Once no clear local side effects or systemic toxic effects are observed, the compound can then progress to the multiple-dose toxicity study.

Study design:

1. Test system:

Species:	Mice
Strain:	CD-1
Source:	Charles River Canada Inc.
Number of Female Mice Ordered:	10
Target Weight at the Initiation of Dosing:	5-6 weeks old females (20-25 g)

1.1. Justification of Test System and Number of Animals

The choice of the animal species and strain are in accordance with the standard conventions to establish a preliminary toxicity profile of our products. The CD1 mice are established experimental mouse strains used for drug screening and evaluation (Vogel HG et al., 2011). Our preliminary toxicity studies has demonstrated that the Compound X (the parent drug) did not have significant toxicity in CD1 mice.

1.2. Animal Identification:

Each animal was identified on the tail for the study number assignment, using indelible ink (permanent marker).

1.3. Environmental Acclimation

A minimum acclimation period of 5 days at the animal facility (INRS) was allowed between animal receipt and the start of treatment in order to accustom the animals to the laboratory environment.

2. Selection, Assignment, and Replacement of Animals

Animals were assigned to groups by randomization designed to achieve similar group mean body weights. Animals in poor health or at extremes of body weight range were not assigned to groups.
We used a free website for randomization: http://www.graphpad.com/quickcalcs/randomize1.cfm

as

The random number generator is seeded with the time of day. Each subject is first assigned to a group non-randomly. Then the assignment of each subject is swapped with the group assignment of a randomly chosen subject. The entire process is repeated twice to make sure it is really random.

Table1: Group assignment for blood collection

Assign subjects to groups *	
Subject #	Group Assigned
1	A
2	B
3	B
4	A
5	B
6	A
7	A
8	B

A= Hematology group
B= Biochemistry group

Table 2: Randomization

After the randomization we excluded the largest and the smallest animal from the study.
Before the initiation of dosing, any assigned animals considered unsuitable for use in the study were replaced by alternate animals obtained from the same shipment and maintained under the same environmental conditions. The disposition of all animals were documented in the study records.

Animal #	Body Weight (g)	Sort by BW		Sort by Arrival #		Animal Arrival #	* Study Group assigment For H/BCH	Animal Study Group #	Animal Arrival Group #	* Study Group assigment	Sort by Animal Study Group #
		Animal arrival #	BW	Arrival	BW						
1	24.56	7	21.80	1	24.56	1	24.56	A	1	24.56	A
2	22.17	8	21.90	2	22.17	2	22.17	B	4	22.57	A
3	22.02	3	22.02	3	22.02	3	22.02	B	6	24.93	A
4	22.57	2	22.17	4	22.57	4	22.57	A	8	21.90	A
5	22.34	5	22.34	5	22.34	5	22.34	B	2	22.17	B
6	24.93	4	22.57	6	24.93	6	24.93	A	3	22.02	B
7	21.80	9	23.07	8	21.90	8	21.90	A	5	22.34	B
8	21.90	1	24.56	9	23.07	9	23.07	B	9	23.07	B
9	23.07	6	24.93								
10	24.99	10	24.99								

	Spare
A=	Hematology Group
B=	Biochemistry Group

3. Food and Water supply

The animals received Teklad global 18% protein diet (cat#:2018) produced by Harlan Laboratories (Montréal, Qc. Canada) and municipal tap water treated by reverse osmosis and UV treatment in a sterile bottle, ad libitum.

4. In-life procedures, observations and measurements

Prior to the experiments, all the animals were acclimatized for 5 days as per the institutional animal care recommendations. The experiment was conducted as a set of pilot studies to screen the in vivo toxicity and pharmacokinetics of our newly developed drugs. One compound was tested to determine their dose ranges, which are tolerated by CD1 mice. A total of 8 mice were used for this study. Each animal was injected with 50 mg/Kg/day of compound every 3 days for a period of 28 days and they were euthanized at day 31.

Group 1 (compound NWL-117Na): 8 animals intraperitoneal administered with a single bolus injection of 50 mg/kg/day of drug concentration.

All animals were received an intra peritoneal injection of 50 mg/kg/day of NWL-117Na compound.

ǿs

4.1. Mortality/Moribundity Checks: (by the INRS staff)
Frequency: Twice daily, once in the morning and once in the afternoon, throughout the study.

4.2. Cage side Observations:
Frequency: Following drug administration, all the animals were monitored for a period of 24 h for any adverse events (once at following time-point ranges: 0-4 h, 6-8h and 21-24 h).

4.3. Detailed clinical observation:
Frequency: Before randomization and treatment

4.4. Body Weights
Frequency: Before randomization, on treatment day 1, 4, 7, 10, 13, 16, 19, 22, 25, 28 and at the end of study on day 31.

Procedure: Animals were individually weighed.

Treatment:

Mice were randomly grouped for NWL-117Na intra peritoneal injection. The animals received 50 mg/Kg/day, dosing volume 6 mL /Kg, on day 1, 4, 7, 10, 13, 16, 19, 22, 25 and 28.

4.5. Compound:
Identification: NWL-117Na (NWL-117Na-04) for Injection, USP

Supplier: New World Laboratories

Batch (Lot) No.: 4

Expiration Date: N/A

Physical Description: Liquid

Purity: 98%

Concentration: 8.33 mg/mL

Storage Conditions: When stored at ambient temperature, the solution should be used within the next 4 hours. The mixture can be stored at +4C for a maximum of 3 days prior its use at ambient temperature. It is suggested to prepare a fresh solution every 3 days. The solution can also be stored at -29°C without deterioration (6 days). Kept in a freezer at -29°C in chemistry lab or in Freezer at -20°C in INRS.

NWL-117 bis-sodium salt (NWL-117Na): Preparation of a solution of 8.33 mg/mL of NWL-117Na in 0.9% saline (1 mL). To a sterile vial containing 8.33 mg of NWL-117Na was added at once 1 mL of 0.9% saline. The mixture was vortexed for 1 minute to give a clear solution.

4.6. Reserve Samples:
For each batch (NWL-117Na-04) of compound, a reserve sample (1 vial of 50 µL) was collected and maintained under the appropriate storage conditions.

5. Terminal procedures:

5.1. Scheduled Euthanasia
All animals were sacrificed on day 31 by exsanguination after blood collection by cardiac puncture after isoflurane anesthesia. The animals were subjected to a complete necropsy examination which included evaluation of the carcass; all external surfaces and orifices; cranial cavity and external surfaces of the brain; and thoracic, abdominal, and pelvic cavities with their associated organs and tissues.

5.2. Unscheduled Deaths
There were no unscheduled deaths.

6. Sample Collection, Processing, and Analysis:

6.1. Blood collection
Blood was collected from select animals on day 31 by cardiac puncture under isoflurane anesthesia for hematology and biochemistry analysis.

6.2. Blood analysis (H/BCH): Idexx

8 samples from treated mice and 2 samples from non-treated mice (control) were sent to IDEXX for analysis:

- 4 mice (treated) and 1 mouse (non-treated) for Hematology analysis: whole blood was analyzed fresh.
  Tube Anticoagulant: EDTA K_2 Microtainer 500 µL (#cat:cabd365974, lot#: 4064163, exp.:2015-08)
  Target Volume: 0,5 mL
  Centrifugation: N/A
  Storage: in wet ice or 4°C

Idexx test code: CCBC: WBC, RBC, Hgb, Hct, erythrocyte indices (MCV, MCH, MCHC), WBC differential, platelet count, reticulocyte count, smear evaluation by a technologist for RBC and WBC morphology and parasite screen, pathologist review of abnormal cells.

- 4 mice (treated) and 1 mouse (non-treated) for Biochemistry analysis:
  Tube: BD Microtainer serum separator tubes (SST) 250-500 µL (#cat:cabd365952, lot# 3305334, exp.:2015-01)
  Target Volume: 0,5 mL
  Centrifugation: incubate at room temperature for 20-30 min. and centrifuged at Room temperature (4 500 Rpm) for 10 min. at 4°C. Serum was transferred into the 0,5 ml Eppendorf tube.
  Storage: room temperature for 4 hours maximum or 4°C

Idexx test code: CHM02: Albumin, albumin/globulin ratio, alkaline phosphatase, ALT (SGPT), amylase, bilirubin (total), calcium, creatinine, globulin, glucose, total protein, urea.

6.3. Shipping Contact

Idexx Laboratories Inc.
Crystal Campeau
Toronto, On, M5W 5W6
T: Mobile: (514) 805-9152
T: 1-866-683-2551
E: crystal-campeau@idexx.com
Vetconnectplus.ca

The laboratory was notified the same day before shipment of the samples before 11:00 AM (to send before 1:00 PM) or before 6:00 PM (to ship before 8:00 PM). Upon receipt at the laboratory, the samples were stored under the following conditions: room temperature for 4 hours maximum or 4°C.

7. Histology:

7.1. Tissue Collection and Preservation

All major tissues and organs were collected and fixed 48 hrs in 1:10 Tissufix (CHAPTEC INC.) at room temperature.

7.2. Histology

Histopatological processing and analysis of all major tissues and organs was performed at McGill University (Goodman cancer research center histology core facility). According to their standard operating procedures, all fixed tissues were placed into cassettes and embedded into paraffin (the sternum was decalcified prior processing). Blocks were cut with a microtome and the sections were stained with Hematoxylin & Eosin (H&E), mounted and examined under a bright field microscope by Comparative Pathology Service in Mc Gill University for histopathological analysis by a certified veterinary pathologist. Each submitted tissue section was evaluated for tissue abnormalities and scored according to the intensity of the observed changes:

The tissues in paraffin block were sent back to New World Laboratories.

Table 3: Major tissue and organ inventory

Organ	Animal#							
	1	2	3	4	5	6	8	9
Skin Rigt injection site	√	√	√	√	√	√	√	√
Skin Left injection site	√	√	√	√	√	√	√	√
Liver	√	√	√	√	√	√	√	√
Spleen	√	√	√	√	√	√	√	√
Pancreas	√	√	√	√	√	√	√	√
Kidney Right	√	√	√	√	√	√	√	√
Heart	√	√	√	√	√	√	√	√
Lungs	√	√	√	√	√	√	√	√
Brain	√	√	√	√	√	√	√	√
Right Femur or sternum	√	√	√	√	√	√	√	√
Eye left (small)					√			
Eye right (normal)					√			

7.3. Contact

Mc Gill
Contact person
Jean-Martin Lapointe, DMV, MSc, dACVP
Comparative Pathologist
Comparative Medicine – Animal Resources Centre
McGill University
3655 Pr. Sir William Osler, rm 1440
Montreal, Qc
Canada H3G 1Y6
Phone: 514 398-4400 (00535)
email: jean-martin.lapointe@mcgill.ca

8. Results and discussion

8.1. : Mortality and clinical observation (cage side observation)

No mice were found dead, and no significant and systemic clinical signs were observed during the study, except for number 5. This animal #5, was found with a left eye smaller then the right eye (microphtalmia); this is usually a birth defect. The mouse has no signs of distress or infection. Eye was sent to the pathologist for analysis at the end of the study.

No visible tumor growth at the site of injection was observed for all groups during the entire 31 days study period.

8.2. Body Weight

Individual body weight were recorded before randomization and every three days on treatment day 1, 4, 7, 10, 13, 16, 19, 22, 25, 28 and at the end of study on day 31. There was no difference between the animals.

Table 3: Body Weight of mice treated with NWL-117Na (50mg/Kg i.p.) for 31 days.

Day	50 mg/Kg
0	22.945
1	22.945
4	23.615
7	23.63
10	24.42
13	25.08
16	25.30
19	25.97
22	26.39
25	27.34
28	27.27
31	26.66

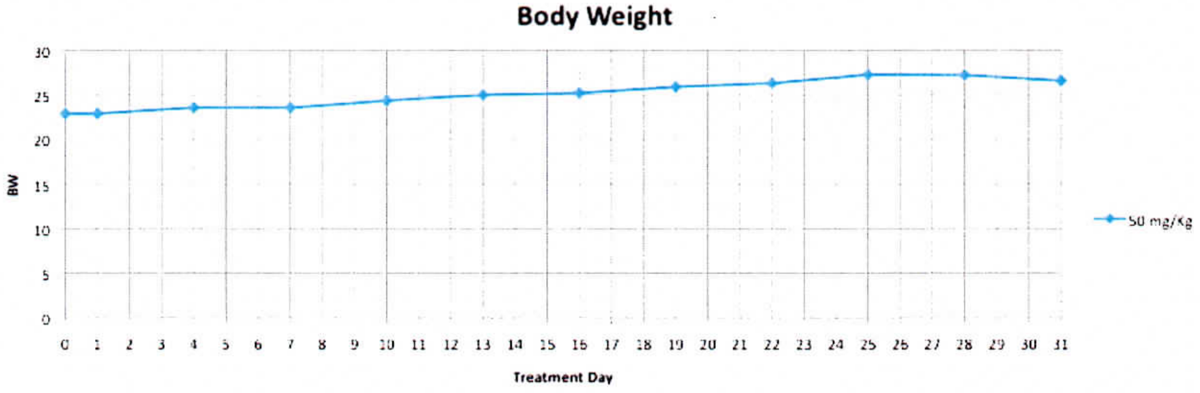

8.3. Hematology and Biochemistry analysis

Blood was collected from selected animals at the end of the study (at day 31). The hematology and Biochemistry results are comparable to the reference range from Charles River (CD-01 Mouse Hematology north American colonies Jan 2008-Dec 2011, 8-10 weeks old), Research animal resources from University of Minnesota, (http://www.ahc.umn.edu/rar/refvalues.html), "Ferrets, rabbits and rodent clinical medicine and surgery" by Katherine Quesenberry and James Carpenter. Animal #9 has a level of amylase lower than normal. It can be related to the severe hepatic dysfunction but decreased amylase is seldom clinically significant. No clinical signs were observed for that animal.

Table 4: Hematology analysis of mice treated with NWL-117Na, non-treated and reference range.

Mouse#	1	4	6	8	Average	Average 28-day tox study on CD-1 mice (NWL-53)	28-day tox study on CD-1 mice (control)	CD-1 Control mouse without injection (12 weeks)	Reference Range
RBC (x10^12/L)	9.5	10	8.6	8.7	9.2	6.01-8.51	9.4-10.3	8.9	7.9-10.1
Hematocrit (L/L)	0.45	0.47	0.4	0.41	0.4325			0.44	0.37-0.46
Hemoglobin (g/L)	148	156	131	128	140.75	105.14-129.26	129.02-153.48	144 (14.4 g/dL)	110-145
MCV (fL)	48	47	47	47	47.25	108.69-128.87	89.53-131.03	49	41-49
MCH (pg)	16	16	15	15	15.5			16	12.9-18.77
MCHC (g/L)	326	333	327	315	325.25	527.44-634.96	418.28-673.72	329	219-335
% reticulocyte	3.1	2.6	2.3	4.5	3.125			3.1	
Reticulocyte (K/uL)	294.5	260	197.8	391.5	285.95			275.9	
WBC (x10^9/L)	8.8	5.3	4.7	4.1	5.725	2.56-3.12	2.76-3.94	5.5	5.0-13.7
% Neutrophil	14.2	13.1	13.5	11.5	13.075			16	6.7-40
% Band	0	0	0	0	0			0	
% Lymphocyte	80.6	81.9	78	83.2	80.925			81.5	55-95
% Monocyte	3.4	1.9	3	1.9	2.55			0.7	0.1-3.5
% Eosinophil	1.7	2.9	5.3	3.4	3.325			1.6	0-4
%Basophil	0.1	0.2	0.2	0	0.125			0.2	0-1.5
Neutrophil (x10^9/L)	1.2	0.7	0.6	0.5	0.75	0.17-0.23	0.23-0.73	0.9	0.54-4.31
Band (x10^9/L)	0	0	0	0	0			0	
Lymphocyte (x10^9/L)	7.1	4.3	3.7	3.4	4.625	1.54-2.08	0.9-1.6	4.5	2.7-11.3
Monocyte (x10^9/L)	0.3	0.1	0.1	0.1	0.15	0.04-0.06	0.08-0.26	0	0.22-1.49
Eosinophil (x10^9/L)	0.1	0.2	0.2	0.1	0.15	0.06-0.1	0.04-0.06	0.1	0.01-0.60
Basophil (x10^9/L)	0	0	0	0	0			0	0-0.16
Platelet (x10^9/L)	1.038	1.131	1.7	1.335	1.301	358.53-489.07	321.59-452.41	1.059	100-1200 $x10^3$/uL
Smear Evaluation									
WBC Morphology	N	N	N	N				N	
RBC Morphology	Polychromasia -6/HPF	Polychromasia -6/HPF	Polychromasia -6/HPF	Polychromasia -6/HPF				Polychromasia -6/HPF	
PLT Morphology	Clumped platelets-marked	Clumped platelets-Mild	N	N				Clumped platelets-Mild	

Table 5: Biochemistry analysis of mice treated with NWL-117Na, non-treated and reference range.

Mouse#	2	3	5	9	Average	Average 28-day tox study on CD-1 mice (NWL-53)	28-day tox study on CD-1 mice (control)	CD-1 Control mouse without injection (12 weeks)	Reference Range
Glucose (mmol/L)	10.7	11.4	10	11.3	10.85	11.32-13.16	8.39-10.41	12.5	9.71-18
BUN (mmol/L)	8.9	7.1	7.8	6.5	7.58	5-6.5	4.43-4.57	6.8	12.14-20.59
Creatinine (umol/L)	15.03	12.38	10.61	13.26	12.82	36	36	10.61	0.2-1.1 mg/dL
Calcium (mmol/L)	2.23	2.21	2.24	2.21	2.22			2.36	4.6-9.6 mg/dL
Total Protein (g/L)	45	50	47	45	46.75	56.13-66.27	57.87-84.13	46	42-103
Albumin (g/L)	26	29	27	27	27.25			27	21-48
Globulin (g/L)	19	21	20	18	19.50			19	18-82
Alb/Glob Ratio	1.4	1.4	1.4	1.5	1.43			1.4	1.23-1.37
ALT (U/L)	27	18	21	24	22.50	41.49-49.71	34	20	17-77
ALP (U/L)	72	121	69	100	90.50	34.71-58.29	72	77	23.7-96
Bilirubin Total (umol/L)	2.39	2.57	2.22	2.22	2.35	6.42-7.58	8	1.54 (0.1 mg/dL)	0.15-0.85 mg/dL
Amylase (U/L)	937	742	674	3.779	589.19			750	
Hemolysis Index	N	N	N	N	N			N	
Icterus Index	N	N	N	N	N			N	
Lipemia Index	N	N	N	N	N			N	

8.4. Necropsy- gross pathology

The necropsy was performed at the end of study. No gross abnormality or tumor growth was observed.

8.5. Necropsy- histopathology

Changes were observed in some sections of lung, liver, pancreas and brain. In the lung of one animal, there were areas of acute hemorrhage with the alveolar lumina. This was considered most likely an agonal change, probably related to euthanasia or possibly terminal blood sampling, but most unlikely to be treatment-related. In the liver, three animals had a few small foci of mixed inflammatory cell infiltration. This is a common background observation in healthy mice, and at this minimal severity-grade it was not considered likely to be related to treatment. In the pancreas, foci of inflammatory cell infiltration (mainly histiocytes and lymphocytes) were observed in the mesentery sheets associated with the pancreas samples. The pancreatic parenchyma itself was not affected. This was considered mostly likely a consequence of inflammation secondary to the intra-peritoneal injection (since animals receiving vehicle-only injections were not submitted, it cannot be concluded whether the inflammatory reaction was secondary to the injection itself, to the vehicle, or to the administered compound). One animal had changes of vacuolation, most likely neuronal-associated, in the cerebral cortex and to a lesser extent the hippocampus. The vacuolation was bilateral, but only noted at one level of the cortex (frontal and dorsal area). This is not a commonly observed or reported background change in mice. It may have been an effect of the compound administered, or a pathologic change unrelated to the compound. The extent of the vacuolation was limited, and it was not associated with other significant changes in the brain.

Histopathology conclusion: The two changes potentially associated with treatment were inflammation in the mesentery, and neuronal vacuolation in the brain. Mesentery inflammation was likely a consequence of irritation related to the intra-peritoneal route of administration itself, but without control groups we cannot conclude whether it was a direct effect of the compound or related to the vehicle or injection. The cerebral vacuolation was observed only in one animal; it may have been a drug effect, or an unrelated problem in this animal. Additional studies would be necessary to conclude as to the possibility of a drug effect.

Pathologist: Jean-Martin Lapointe, DMV, MSc, dACVP

Table 7: Pathology results

ID	Spleen	Kidney	Heart	Skin (right side)	Skin (left side)	Sternum (bone marrow)	Lungs	Liver	Pancreas	Brain
1	wnl	wnl	wnl	wnl	wnl	wnl	Hemorrhage, alveolar, acute	Inflammatory infiltration, mixed, minimal	wnl	wnl
2	wnl	wnl	wnl	wnl	wnl	wnl	wnl	wnl	wnl	wnl
3	wnl	wnl	wnl	wnl	wnl	wnl	wnl	wnl	Inflammation, histiolymphocytic, mesentery, minimal	wnl
4	wnl	wnl	wnl	wnl	wnl	wnl	wnl	wnl	wnl	wnl
5	wnl	wnl	wnl	wnl	wnl	wnl	wnl	wnl	wnl	wnl
6	wnl	wnl	wnl	wnl	wnl	wnl	wnl	wnl	Inflammation, histiolymphocytic, mesentery, minimal	wnl
8	wnl	wnl	wnl	wnl	wnl	wnl	wnl	Inflammatory infiltration, mixed, minimal	Inflammation, histiolymphocytic, mesentery, mild	wnl
9	wnl	wnl	wnl	wnl	wnl	wnl	wnl	Inflammatory infiltration, mixed, minimal	Inflammation, histiolymphocytic, mesentery, mild	Neuronal vacuolation, cortex and hippocampus, bilateral, focal, mild

wnl: within normal limits

9. Conclusion

CS

The primary purpose of this study was to evaluate if NWL-117Na at 50-mg/Kg intraperitoneal injection has any local reaction and toxicity. None of the injected animals formed tumors but some of them had an inflammation reaction in the mesentery. One animal has changes of vacuolation in the cerebral cortex and to a lesser extent in the hippocampus and the level of Amylase was lower than normal. This could be, or not, associated with the injection of 50 mg/Kg of NWL-117Na.

10. Appendix

10.1. Appendix #1: Pathology report (appendix in a separate document).

Cs

11. Signature

Report Approval

Report Prepared by:	Annie Salesse
Title	Research Associate
Signature / Date (YYYY-MM-DD)	Annie Salesse 2014-09-03
**Report Reviewed and Approved by**	**Jan-Eric Ahlfors**
Title	CEO-CSO
Signature / Date (YYYY-MM-DD)	[illegible] 2014-09-03

Comparative Medicine
Animal Resources Centre

McGill

Comparative Pathology Service
tel: (514) 398-4400 xt 00535
e-mail: jean-martin.lapointe@mcgill.ca

Pathology Report

Submitter/Investigator: Annie Salesse / New World Laboratories

Date issued: August 20th 2014

Date submitted: July 8th 2014

Objectives: Evaluate tissues in mice administered an unspecified therapeutic compound

Samples: Information from Sponsor: Mice were randomly grouped for NWL-117Na intraperitoneal injection. The animals received 50 mg/kg/day, dosing volume 6 mL/kg, on day 1, 4, 7, 10, 13, 16, 19, 22, 25 and 28. Animals were sacrificed at day 31.

Samples of liver, spleen, pancreas, skin injection sites (left and right), kidney, heart, lungs, brain, sternum and femur from 8 mice were submitted in formalin. All tissues except the femur were processed for histology. The container for animal #5 was indicated via separate email to additionally contain 2 eyes, but these were not noticed at trimming, and may have been lost. The sternum was decalcified prior to processing. Sections were stained with hematoxylin and eosin and examined by the pathologist.

Results / Discussion: see Excel table in separate document for details.

Changes were observed in some sections of lung, liver, pancreas and brain.

In the lung of one animal, there were areas of acute hemorrhage with the alveolar lumina. This was considered most likely an agonal change, probably related to euthanasia or possibly terminal blood sampling, but most unlikely to be treatment-related.

In the liver, three animals had a few small foci of mixed inflammatory cell infiltration. This is a common background observation in healthy mice, and at this minimal severity grade it was not considered likely to be related to treatment.

In the pancreas, foci of inflammatory cell infiltration (mainly histiocytes and lymphocytes) were observed in in the mesentery sheets associated with the pancreas samples. The pancreatic parenchyma itself was not affected. This was considered mostly likely a consequence of inflammation secondary to the intra-peritoneal injection. Since animals receiving vehicle-only injections were not submitted, it cannot be concluded whether the inflammatory reaction was secondary to the injection itself, to the vehicle, or to the administered compound.

One animal had changes of vacuolation, most likely neuronal-associated, in the cerebral cortex and to a lesser extent the hippocampus. The vacuolation was bilateral, but only noted at one level of the

LIMITATION OF PROFESSIONAL LIABILITY & WARRANTY
The Investigator agrees to limit any and all liability or claim for damages, for cost of defense, or for any expenses to be levied against McGill University and the Animal Resources Centre to a sum not to exceed $1,000 or the amount of the fee for diagnostic or technical services performed, whichever is less when such claim arises from any error, omission, or professional negligence on the part of the Animal Resources Centre. Professional services performed, findings obtained and recommendations prepared are in accordance with generally and currently accepted consulting principles and practices in laboratory animal medicine and regulations and guidelines for the care and use of laboratory animals. This warranty is in lieu of all other warranties either expressed or implied

Comparative Medicine
Animal Resources Centre

McGill

Comparative Pathology Service
tel: (514) 398-4400 xt 00535
e-mail: jean-martin.lapointe@mcgill.ca

Pathology Report

cortex (frontal and dorsal area). This is not a commonly observed or reported background change in mice. It may have been an effect of the compound administered, or a pathologic change unrelated to the compound. The extent of the vacuolation was limited, and it was not associated with other significant changes in the brain.

Conclusion:

The two changes potentially associated with treatment were inflammation in the mesentery, and neuronal vacuolation in the brain. Mesentery inflammation was likely a consequence of irritation related to the intra-peritoneal route of administration, but without control groups we cannot conclude whether it was a direct effect of the compound or related to the vehicle or injection.

The cerebral vacuolation was observed only in one animal; it may have been a drug effect, or an unrelated problem in this animal. Additional studies would be necessary to conclude as to the possibility of a drug effect.

It should be noted that any study evaluating possible toxicity of a compound should always include a control group, treated with the drug vehicle only, at the same volume and frequency as the test groups, and kept in the same conditions. This is absolutely necessary in order to be able to adequately interpret any pathologic finding observed.

(joined: Excel document with results).

Pathologist: Jean-Martin Lapointe, DMV, MSc, dACVP

LIMITATION OF PROFESSIONAL LIABILITY & WARRANTY
The Investigator agrees to limit any and all liability or claim for damages, for cost of defense, or for any expenses to be levied against McGill University and the Animal Resources Centre to a sum not to exceed $1,000 or the amount of the fee for diagnostic or technical services performed, whichever is less, when such claim arises from any error, omission, or professional negligence on the part of the Animal Resources Centre. Professional services performed, findings obtained and recommendations prepared are in accordance with generally and currently accepted consulting principles and practices in laboratory animal medicine, and regulations and guidelines for the care and use of laboratory animals. This warranty is in lieu of all other warranties either expressed or implied.

Appendix #1

Comparative Pathology Service
tel: (514) 398-4400 xt 00535
e-mail: jean-martin.lapointe@mcgill.ca

Pathology results

McGill University

ID	Spleen	Kidney	Heart	Skin (right side)	Skin (left side)	Sternum (bone marrow)	Lungs	Liver	Pancreas	Brain
1	wnl	wnl	wnl	wnl	wnl	wnl	Hemorrhage, alveolar, acute	Inflammatory infiltration, mixed, minimal	wnl	wnl
2	wnl	wnl	wnl	wnl	wnl	wnl	wnl	wnl	wnl	wnl
3	wnl	wnl	wnl	wnl	wnl	wnl	wnl	wnl	Inflammation, histiolymphocytic, mesentery, minimal	wnl
4	wnl	wnl	wnl	wnl	wnl	wnl	wnl	wnl	wnl	wnl
5	wnl	wnl	wnl	wnl	wnl	wnl	wnl	wnl	wnl	wnl
6	wnl	wnl	wnl	wnl	wnl	wnl	wnl	wnl	Inflammation, histiolymphocytic, mesentery, minimal	wnl
8	wnl	wnl	wnl	wnl	wnl	wnl	wnl	Inflammatory infiltration, mixed, minimal	Inflammation, histiolymphocytic, mesentery, mild	wnl
9	wnl	wnl	wnl	wnl	wnl	wnl	wnl	Inflammatory infiltration, mixed, minimal	Inflammation, histiolymphocytic, mesentery, mild	Neuronal vacuolation, cortex and hippocampus, bilateral, focal, mild

wnl: within normal limits

As for
Jean-Martin Lapointe
pathologist from
McGill university
2014-09-03
