# Supplementary material for: Caspase vinyl sulfone small molecule inhibitors prevent axonal degeneration in human neurons and reverse cognitive impairment in Caspase-6-overexpressing mice
Source: Mol Neurodegener. 2017 Feb 28;12:22. doi: 10.1186/s13024-017-0166-z (PMC5329948; doi:10.1186/s13024-017-0166-z)
Supplement: Additional file 2: Figures S1-S4. — Contains additional information to complement Figs. 3, 4, and 6 of the manuscript. (PDF 463 kb) [file 13024_2017_166_MOESM2_ESM.pdf]

Additional file 2

Caspase vinyl sulfone small molecule inhibitors prevent axonal degeneration in human neurons and reverse cognitive impairment in Caspase-6-overexpressing mice.

Prateep Pakavathkumar[1,2], Anastasia Noël[1,2], Clotilde LeBlond-Lecrux[3], Agne Tubeleviciute-Aydin[1,2], Edith Hamel[3], Jan-Eric Ahlfors[4], Andrea C. LeBlanc[1,2]

[1] Bloomfield Center for Research in Aging, Lady Davis Institute for Medical Research, Jewish General Hospital, 3755 Ch. Cote Ste-Catherine, Montreal, Quebec, Canada H3T 1E2

[2] Department of Neurology and Neurosurgery, McGill University, 3775 University St., Montreal, QC, Canada H3A 2B4

[3] Laboratory of Cerebrovascular Research, Montreal Neurological Institute, McGill University, Montreal, Quebec, Canada H3A 2B4

[4] New World Laboratories, 500 Boulevard Cartier Ouest, Laval, Quebec, Canada H7V 5B7

Short title: NWL Caspase-6 inhibitors

Corresponding author: Andrea LeBlanc, PhD, Molecular and Regenerative Medicine Axis, Lady Davis Institute for Medical Research, Sir Mortimer B Davis Jewish General Hospital, 3755 ch. Côte Ste-Catherine, Montréal, QC, Canada H3T 1E2. Tel.: +1 (514) 340 8222 ext 4976. Fax.: +1 (514) 340 8295. e-mail address: andrea.leblanc@mcgill.ca

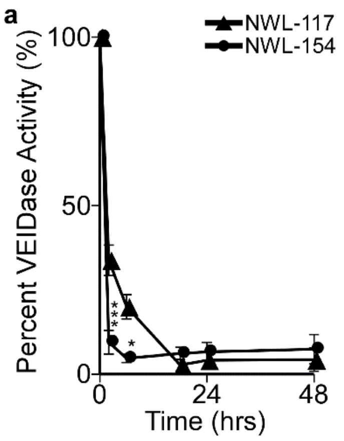

Supplemental Figure S1. (**a**) Percent VEIDase activity from cellular protein extracts of pCep4β-Casp6p20p10-transfected HCT116 cells treated with either PEG-soluble NWL-117 (closed triangle) or -154 (closed circle) at 100 μM for 0, 2, 6, 18, 24, or 48 hours. Data represent the mean ± SEM of three independent experiments. Statistical analysis was performed by two-way ANOVA (compound ($p = 0.0240$), time ($p < 0.0001$), interaction ($p = 0.0018$)) with Bonferroni post tests (* $p < 0.05$, *** $p < 0.001$).

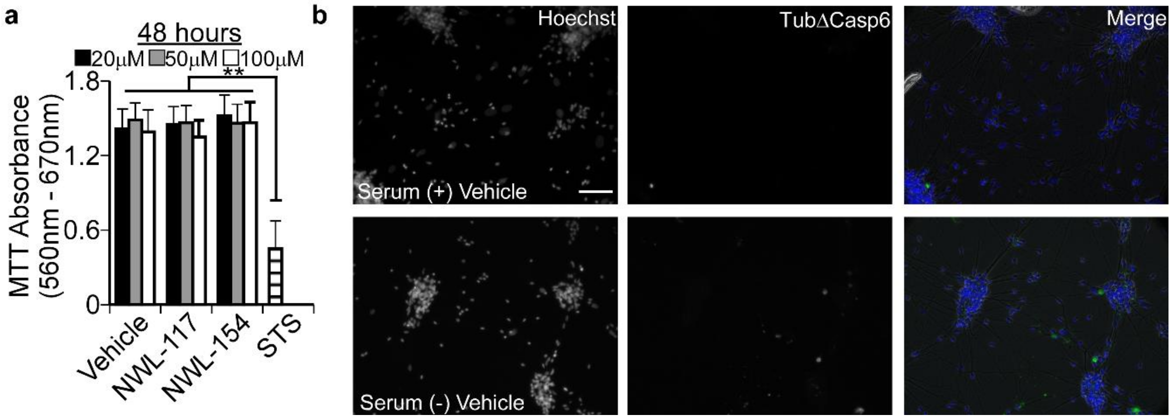

Supplemental Figure S2. (**a**) MTT absorbance from human neurons treated with PBS vehicle, water-soluble NWL-117, or NWL-154 at 20, 50, or 100 μM or 2 μM staurosporine for 48 hours. Data represent the mean ± SEM of at least three independent experiments. Statistical analysis was done by one-way ANOVA with Tukey's multiple comparison test ($p = 0.0024$) (* compares to staurosporine treatment: ** $p < 0.01$) unless stated otherwise. (**b**) Phase contrast and fluorescence micrographs of human neurons transfected with pBudEGFP or pBudEGFP/APP^{WT} treated with vehicle and stained for α-tubulin-cleaved by Casp6 (TubΔCasp6) (Alexa 488) and Hoechst before or after serum-deprivation. Scale bar represents 100 μm.

Supplemental Figure S3. Pakavathkumar et al., NWL Caspase-6 inhibitors

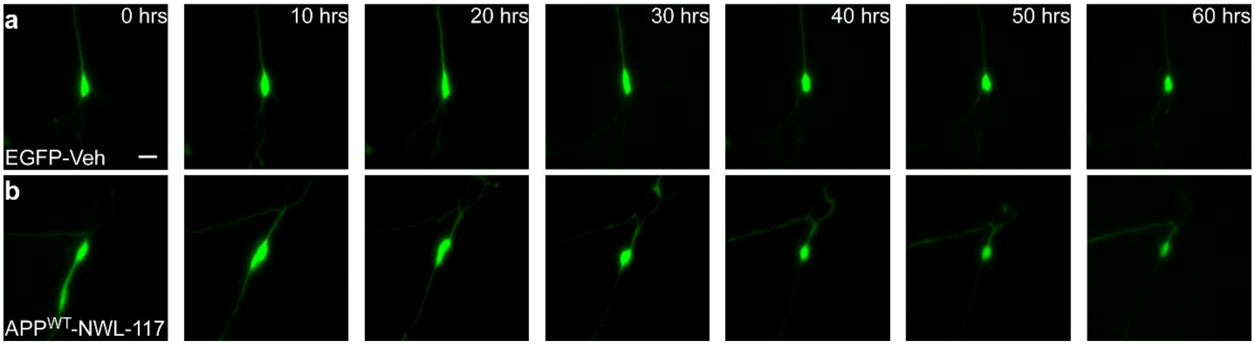

Supplemental Figure S3 (**a & b**) Live imaging fluorescence micrographs at 0, 10, 20, 30, 40, 50, and 60 hours of a human neuron transfected with pBudEGFP or pBudEGFP/APP^{WT}. Primary human neurons were pre-treated with vehicle (**a**) or 100 μM water-soluble NWL-117 (**b**) starting 2 hours prior to transfection. Scale bar represents 10 μm.

Supplemental Figure S4. Pakavathkumar et al., NWL Caspase-6 inhibitors

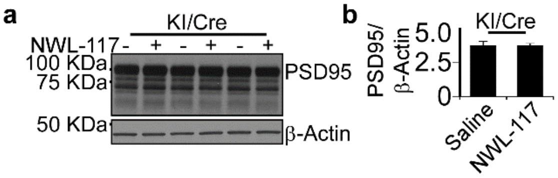

Supplemental figure S4 (**A & B**) Levels of PSD95 in hippocampal extracts from KI/Cre mice treated with saline (n=3) or 20 mg/Kg water-soluble NWL-117 (n=3) (**a**) and quantified in (**b**). For panel B, data represent the mean ± SEM. Statistical analysis was performed by unpaired two-tailed t test and no significant differences were found.
